# Improved GGIW-PHD filter for maneuvering non-ellipsoidal extended targets or group targets tracking based on sub-random matrices

**DOI:** 10.1371/journal.pone.0192473

**Published:** 2018-02-14

**Authors:** Zhibing Liang, Fuxian Liu, Jiale Gao

**Affiliations:** Air and Missile Defense College, Air Force Engineering University, Xi’an, Shaanxi, P.R. China; Southwest University, CHINA

## Abstract

For non-ellipsoidal extended targets and group targets tracking (NETT and NGTT), using an ellipsoid to approximate the target extension may not be accurate enough because of the lack of shape and orientation information. In consideration of this, we model a non-ellipsoidal extended target or target group as a combination of multiple ellipsoidal sub-objects, each represented by a random matrix. Based on these models, an improved gamma Gaussian inverse Wishart probability hypothesis density (GGIW-PHD) filter is proposed to estimate the measurement rates, kinematic states, and extension states of the sub-objects for each extended target or target group. For maneuvering NETT and NGTT, a multi-model (MM) approach based GGIW-PHD (MM-GGIW-PHD) filter is proposed. The common and the individual dynamics of the sub-objects belonging to the same extended target or target group are described by means of the combination between the overall maneuver model and the sub-object models. For the merging of updating components, an improved merging criterion and a new merging method are derived. A specific implementation of prediction partition with pseudo-likelihood method is presented. Two scenarios for non-maneuvering and maneuvering NETT and NGTT are simulated. The results demonstrate the effectiveness of the proposed algorithms.

## Introduction

In traditional multi-target tracking applications, most of the tracking approaches make the assumption that each target can at most generate one measurement at a given time step [1–2], owing to limited sensor resolution and large sensor measurement error relative to the target size. Under this assumption, the kinematic states (e.g., position, velocity, and acceleration) of the targets can be estimated, however, ignoring the extension information (e.g., size, shape, and orientation). With the development of sensor technology, a fluctuating number of measurements can be obtained from a target at an instant time, and such measurements may be only partially resolvable. Direct application of traditional tracking approaches treating a target as a point source will lead to deteriorating tracking results [3–4]. In this case, a target is preferably treated as an extended target with size, shape, and orientation. Besides, for a group of closely-spaced targets in formation where two or more targets may be unresolvable, traditional tracking approaches face similar challenges [5]. For an extended target or target group, knowing size, shape, and orientation information can be useful for identification and classification in practical tracking applications. Thus, there is an increasing need for extended targets and group targets tracking approaches which can estimate both the kinematic and extension states. Actually, when treated as a whole or without considering the individual properties of each target within the group, a target group can be regarded as an extended target [6]. Thus, the following discussions will be concentrated on extended targets tracking (ETT), and obtained approaches for ETT can be applied to group targets tracking (GTT) with little modification.

Modeling the target extension by using a spatial probability distribution, Gilholm and Salmond [7] proposed a novel ETT approach in 2005. This approach assumes that extended target measurements stem from a certain area with high density, and the number of measurements yields to Poisson distribution. An inhomogeneous Poisson point process measurement model is suggested in [8–9], where extended target measurements can be modeled as an ellipsoid approximately.

With finite set statistics (FISST), Mahler presented probability hypothesis density (PHD) filter [10], which can effectively avoid data association problem existed in traditional multi-target tracking algorithms. PHD filter can track an unknown number of multiple targets, in the presence of Poisson false alarms, missed detections, and appearance, disappearance, and spawning of targets. Several other advanced algorithms based on FISST, such as cardinalized PHD (CPHD) filter, cardinality-balanced multi-target multi-Bernoulli (CBMeMBer) filter, and generalized labeled multi-Bernoulli (GLMB) filter, are given in [11–13]. The extended target PHD, CPHD, and CBMeMBer filters, respectively called ET-PHD, ET-CPHD, and ET-CBMeMBer filters, have been presented in [14–16]. However, the above-mentioned ETT filters are only capable of estimating the kinematic states of the targets’ centroids, which may lead to some tracking drawbacks because of the loss of extension information.

To jointly estimate the kinematic and extension states, Koch [17] introduced a random matrix approach in which the extension as an additional random variable is used to characterize the states of extended targets or group targets. The kinematic states are modeled by using a Gaussian distribution, while the extension state is modeled by using an inverse Wishart distribution. Modifications to Koch’s model have been discussed in [18], and a new prediction-update process is given in [19]. Granström presented a Gaussian inverse Wishart PHD (GIW-PHD) filter [20] which incorporates the random matrix approach [17] into PHD framework. Considering the estimation of the measurement rate [21], a gamma GIW-PHD (GGIW-PHD) filter is presented in [22], where the measurement rate is modeled by using a gamma distribution. To achieve performance improvements, GGIW-CPHD and GGIW-GLMB filters have been proposed in [23–24]. However, these filters based on random matrix approach are only applicable to some tracking scenarios where the target extensions can be adequately approximated by an ellipsoid.

Another target extension modeling method is random hypersurface model [25] (RHM), which assumes that varying measurement sources are selected from the hypersurfaces that are scaled versions of the shape boundaries. The target extension can be described more detailedly by star-convex RHM [26]. However, many actual extensions may not be approximated accurately by a star-convex shape [27]. Also, accurate estimations to various types of target extensions are dependent on the corresponding predetermined mathematical equations. With inadequate equations, deteriorating estimation results will be obtained.

For non-ellipsoidal (NE) extended target tracking (NETT), Lan [27] proposed to approximate a NE extension by multiple ellipsoidal sub-objects, each represented by a random matrix. More detailed extension information about size, shape, and orientation can be easily obtained. Then NETT is converted to the estimation of the kinematic and extension states of the sub-objects. For maneuvering NETT (MNETT), the corresponding multi-model (MM) approach based on model combination is discussed in [27]. However, Lan’s approaches are only applicable to a single-target scene without clutter. Besides, these approaches, to the best of our knowledge, have not been used in a framework for tracking an unknown number of extended targets in the presence of missed detections and clutter except [28], where an extended CBMeMBer filter is proposed for NETT problem. However, the approach of [28] only applies to non-maneuvering tracking scenes. Additionally, in its measurement set partitioning method, if a measurement falls into two or more prediction gates, it is only put into the subset corresponding to the prediction component with highest weight, which, however, may discard the correct partitions.

In view of the above-mentioned discussions, this paper makes the following contributions.

1) An improved GGIW-PHD filter based on sub-random matrices is presented to track an unknown number of non-ellipsoidal extended targets. For MNETT, a MM-GGIW-PHD filter, which jointly models both the common and the individual dynamics of the sub-objects belonging to the same extended target or target group through the model combination method, is proposed.

2) For the merging of updating GGIW components, an improved merging criterion based on Hellinger distance is derived. The existing calculation methods in [21,29], etc., only can obtain the individual merging criterions for different state variables by using Kullback-Leibler divergence, which, however, is boundless. In our work, the merging criterion ranging from 0 to 1 is bounded, and it is an integrated whole for all state variables.

3) A new merging scheme for updating GGIW components is derived by moment matching method, instead of using the weighted mean of components as the merging result as in [22,28], etc. The details of the derivation are given in S2 File.

4) For partitioning the measurement set, a specific implementation of prediction partition with pseudo-likelihood method is presented. It can accurately obtain all feasible partitions and select the partitions with *η* largest probabilities through the pseudo-likelihood method.

The rest of this paper is organized as follows. In next section we model the extended target extension based on sub-random matrices approach, and the NETT models are presented in Section 3. Section 4 briefly reviews the ET-PHD filter, and the implementations of the proposed GGIW-PHD filter and MM-GGIW-PHD filter are respectively given in Section 5 and Section 6. The measurement set partitioning method is presented in Section 7, while the simulation results are given in Section 8, before the Conclusion.

## Modeling the extended target extension based on sub-random matrices approach

The extended targets considered in this paper are those sufficiently far away from the sensor so that their measurements resemble a cluster of reflection points spreading over their extensions. When treated as a whole to track, a target group is regarded as an extended target here.

**Fig 1:**
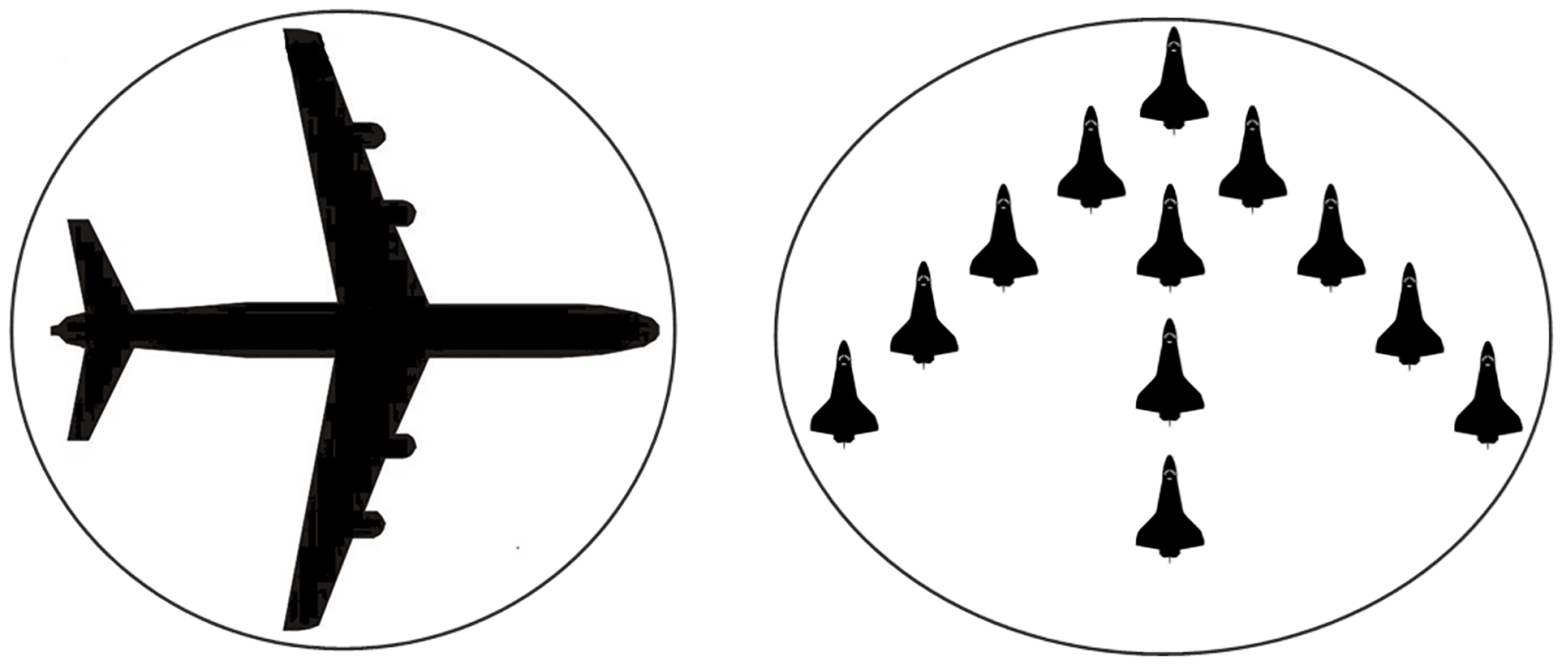
Illustration of approximating an extended target using an ellipse.

It may be not accurate to use an ellipsoid to approximate a NE target extension because of missing the information about shape and orientation. As shown in Fig 1, the ellipse is almost a circle, so that the shapes and orientations of the extended targets cannot be identified.

**Fig 2:**
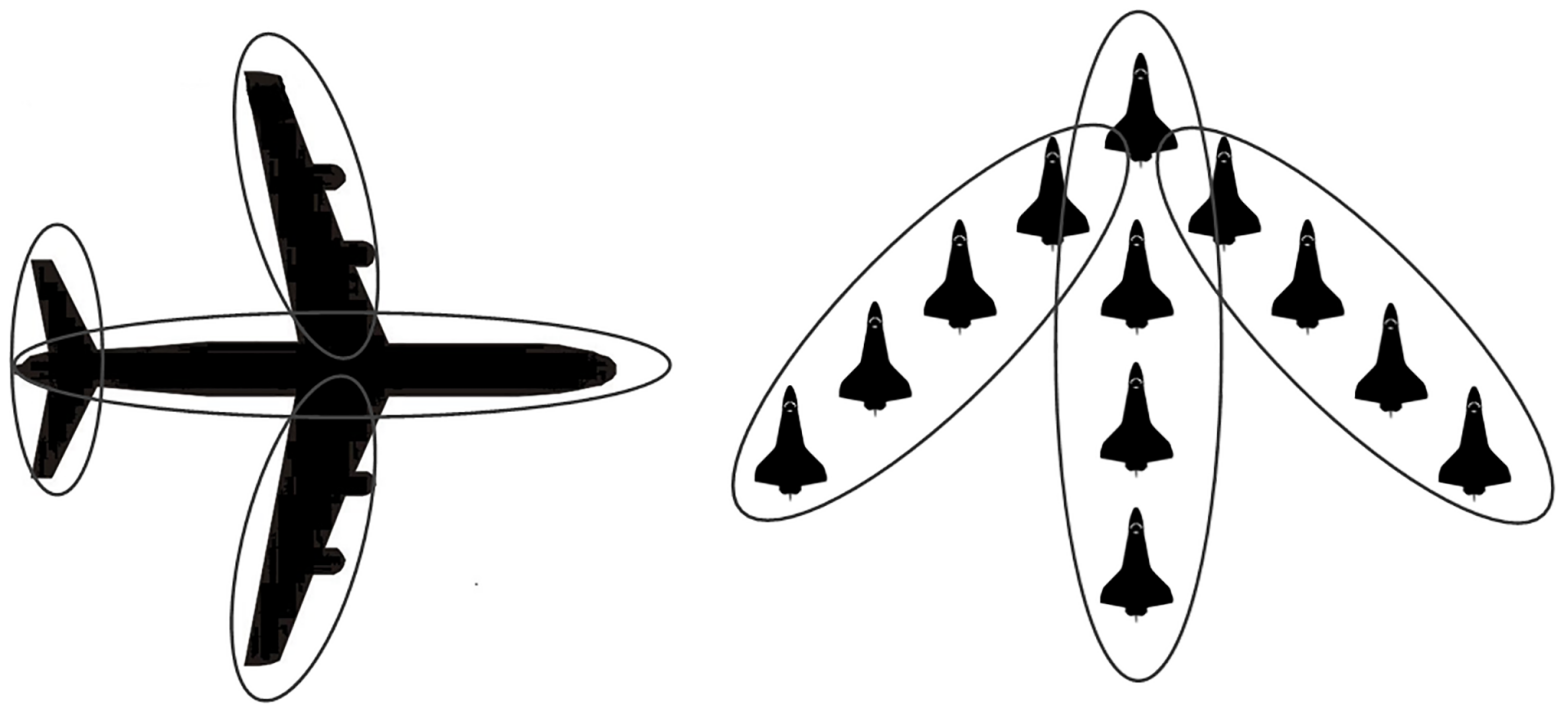
Illustration of approximating an extended target using multiple ellipses.

In this paper, we approximate the target extension by multiple ellipsoids, based on which more detailed information about size, shape, and orientation can be obtained, as illustrated in Fig 2. In this case, sub-objects belonging to the same extended target share kinematic dynamics but have different extension evolution models and initial parameters.

Suppose that $n_{k}^{s}$ is the number of ellipsoidal sub-objects of a NE extended target. The larger $n_{k}^{s}$ is, the more measurements are required to estimate the sub-objects accurately. The selection of $n_{k}^{s}$ is usually not difficult, because a small $n_{k}^{s}$ may be enough to approximate a practical extended target, as shown in Fig 2. Generally, $n_{k}^{s}$ is time-varying to approximate the true extension. Here, it is assumed that $n_{k}^{s}$ is determined and constant, and initial parameters for each sub-object are given.

## NETT models

In this section, we present models for NE extended target state, measurements, and dynamic evolution based on sub-random matrices.

### Notation

- ℝ^*n*×*n*^ is the set of real *n*×*n*-matrices, ℝ^*n*^ is the set of real *n*-vectors, $S_{++}^{n}$ is the set of symmetric positive definite *n*×*n* -matrices, and $S_{+}^{n}$ is the set of symmetric positive semi-definite *n*×*n*-matrices.
- *δ*_*m*,*n*_ is the Kronecker delta, and ⊗ represents the Kronecker product.
- *f*[*g*] denotes the integral ∫ *f*(*x*)*g*(*x*)*dx*.
- For a matrix *A*, |*A*| denotes its determinant. For a set *B*, |*B*| denotes its cardinality.
- G(γ;α,β) denotes a gamma probability density function (pdf) defined over scalar *γ* > 0
  $$G(\gamma ;\alpha ,\beta )=\frac{\beta^{\alpha }}{\Gamma (\alpha )}\gamma^{\alpha -1}e^{-\beta \gamma }$$ (1)
  where scalar shape parameter *α >* 0, scalar inverse scale parameter *β >* 0, and Γ(·) stands for the gamma function. The expected value and variance of *γ* are *α* / *β* and *α* / *β*^*2*^ respectively.
- N(x;m,P) denotes a multi-variate Gaussian pdf defined over the vector $x\in {\mathbb{R}}^{n_{x}}$
  $$N(x;m,P)=\frac{1}{\sqrt{\left|2\pi P\right|}}\text{exp}[-\frac{1}{2}{\left(x-m\right)}^{\text{T}}P^{-1}(x-m)]$$ (2)
  where $m\in {\mathbb{R}}^{n_{x}}$ is the mean vector, and $P\in S_{+}^{n_{x}}$ is the covariance matrix.
- $W_{d}(X;\omega ,W)$ denotes a Wishart pdf defined over the matrix $X\in S_{++}^{d}$ with scalar degrees of freedom *ω* ≥ *d* and parameter matrix $W\in S_{++}^{d}$,
  $$W_{d}(X;\omega ,W)=\frac{2^{-\omega d/2}{\left|X\right|}^{(\omega -d-1)/2}}{\Gamma_{d}(\omega /2){\left|W\right|}^{\omega /2}}etr(-\frac{1}{2}W^{-1}X)$$ (3)
  where *etr*(·) = exp(*tr*(·)) denotes the exponential of the matrix trace, and Γ_*d*_(·) is the multi-variate gamma function. The expected value of *X* is *ωW*.
- ${IW}_{d}(X;v,V)$ denotes an inverse Wishart pdf defined over the matrix $X\in S_{++}^{d}$ with scalar degrees of freedom *ν* ≥ 2*d* and parameter matrix $V\in S_{++}^{d}$,
  $${IW}_{d}(X;v,V)=\frac{2^{-(v-d-1)d/2}{\left|V\right|}^{(v-d-1)/2}}{\Gamma_{d}\left((v-d-1)/2\right){\left|X\right|}^{v/2}}etr(-\frac{1}{2}X^{-1}V)$$ (4)
  The expected value of *X* is *V* / (*ν* − 2*d* − 2).

### Extended target state

Suppose at time *k*, a set of extended targets is denoted by

$$X_{k}={\left\{{\left\{\xi_{k}^{\left(i,s\right)}\right\}}_{s=1}^{n_{k}^{\left(i,s\right)}}\right\}}_{i=1}^{N_{x,k}},\xi_{k}^{\left(i,s\right)}≜(\gamma_{k}^{\left(i,s\right)},x_{k}^{\left(i,s\right)},X_{k}^{\left(i,s\right)})$$ (5)

where *N*_*x*,*k*_ is the unknown number of extended targets, $\gamma_{k}^{\left(i,s\right)}>0$, $x_{k}^{\left(i,s\right)}\in {\mathbb{R}}^{S\times d}$, and $X_{k}^{\left(i,s\right)}\in S_{++}^{d}$ are respectively referred to as the measurement rate, kinematic state, and extension state of sub-object *s* of *i*th extended target, and *S* denotes that the target kinematics are modeled up to (*S* − 1)th derivative. Here, we set *S* = 3. Also, we assume that the number of measurements is Poisson distributed with a gamma distributed parameter $\gamma_{k}^{\left(i,s\right)}$, as defined in [21–23].

Conditioned on previous sequences of measurement sets **Z**^*k*^, the sub-object state $\xi_{k}^{\left(i,s\right)}$ is modeled as a GGIW distribution

$$\begin{array}{lll} p(\xi_{k}^{\left(i,s\right)}|Z^{k}) & =p(\gamma_{k}^{\left(i,s\right)}|Z^{k})p(x_{k}^{\left(i,s\right)}|X_{k}^{\left(i,s\right)},Z^{k})p(X_{k}^{\left(i,s\right)}|Z^{k}) & \left(6\text{a}\right)\\ & =G(\gamma_{k}^{\left(i,s\right)};\alpha_{k|k}^{\left(i,s\right)},\beta_{k|k}^{\left(i,s\right)})N(x_{k}^{\left(i,s\right)};m_{k|k}^{\left(i,s\right)},P_{k|k}^{\left(i,s\right)}⊗X_{k}^{\left(i,s\right)}){IW}_{d}(X_{k}^{\left(i,s\right)};v_{k|k}^{\left(i,s\right)},V_{k|k}^{\left(i,s\right)}) & \left(6\text{b}\right)\\ & =GGIW(\xi_{k}^{\left(i,s\right)};\zeta_{k|k}^{\left(i,s\right)}) & \left(6\text{c}\right) \end{array}$$

where $\zeta_{k|k}^{\left(i,s\right)}=\{\alpha_{k|k}^{\left(i,s\right)},\beta_{k|k}^{\left(i,s\right)},m_{k|k}^{\left(i,s\right)},P_{k|k}^{\left(i,s\right)},v_{k|k}^{\left(i,s\right)},V_{k|k}^{\left(i,s\right)}\}$ is the set of GGIW parameters. Here, there is an implicit assumption that measurement rate $\gamma_{k}^{\left(i,s\right)}$ is independent of $x_{k}^{\left(i,s\right)}$ and $X_{k}^{\left(i,s\right)}$. Actually, the value of $\gamma_{k}^{\left(i,s\right)}$ is dependent on the size of the target and the distance between the sensor and the target. However, it is difficult to model this dependence, which greatly promotes the derivation of model (6).

Estimates of the kinematic state covariance and of the target extension are given by [18]

$${\hat{P}}_{k|k}^{\left(i,s\right)}=\frac{P_{k|k}^{\left(i,s\right)}⊗V_{k|k}^{\left(i,s\right)}}{v_{k|k}^{\left(i,s\right)}+S-Sd-2}$$ (7)

$${\bar{X}}_{k|k}^{\left(i,s\right)}=V_{k|k}^{\left(i,s\right)}/(v_{k|k}^{\left(i,s\right)}-2d-2)$$ (8)

### Measurement model

Assuming that at time *k*, the set of measurements is denoted by

$$Z_{k}={\left\{z_{k}^{\left(j\right)}\right\}}_{j=1}^{N_{\text{z},k}}$$ (9)

where *N*_***z***_,_*k*_ is the number of measurements. The measurement model of sub-object *s* of *i*th extended target is given by [6]

$$\begin{array}{c} z_{k}^{\left(j\right)}=(H_{k}^{s}⊗I_{d})x_{k}^{\left(i,s\right)}+e_{k}^{\left(j,s\right)}\\ e_{k}^{\left(j,s\right)}∼N(e_{k}^{\left(j,s\right)};0,B_{k}^{\left(i,s\right)}X_{k}^{\left(i,s\right)}{\left(B_{k}^{\left(i,s\right)}\right)}^{\text{T}}) \end{array}$$ (10)

where $H_{k}^{s}=[1\;0\;0]$, ***I***_*d*_ ∈ ℝ^d×d^ denotes an identity matrix, $e_{k}^{\left(j,s\right)}$ denotes Gaussian measurement noise with covariance $B_{k}^{\left(i,s\right)}X_{k}^{\left(i,s\right)}{\left(B_{k}^{\left(i,s\right)}\right)}^{\text{T}}$ which can describe the distortion of the observed extension from the actual one, and $B_{k}^{\left(i,s\right)}$ is approximately defined as

$$B_{k}^{\left(i,s\right)}={\left(\lambda {\bar{X}}_{k|k-1}^{\left(i,s\right)}+R_{k}\right)}^{1/2}{\left({\bar{X}}_{k|k-1}^{\left(i,s\right)}\right)}^{-1/2}$$ (11)

where ***R***_*k*_ is the covariance of the true measurement noise, and

$${\bar{X}}_{k|k-1}^{\left(i,s\right)}\approx V_{k|k-1}^{\left(i,s\right)}/(v_{k|k-1}^{\left(i,s\right)}-2d-2)$$ (12)

The number of clutter measurements yields to Poisson distribution with rate *λ*_*k*_, and the clutter measurements are modeled as being uniformly distributed over the surveillance area.

### Dynamic evolution models

The evolution of each extended target is assumed to be independent of other targets here. Also, the state transition density of sub-object *s* of *i*th extended target satisfies

$$p(\xi_{k}^{\left(i,s\right)}|\xi_{k-1}^{\left(i,s\right)})\approx p^{\gamma }(\gamma_{k}^{\left(i,s\right)}|\gamma_{k-1}^{\left(i,s\right)})p^{x}(x_{k}^{\left(i,s\right)}|x_{k-1}^{\left(i,s\right)}\text{,}X_{k}^{\left(i,s\right)})p^{X}(X_{k}^{\left(i,s\right)}|X_{k-1}^{\left(i,s\right)})$$ (13)

For more detailed discussion on state transition density modeling, see, e.g., [17,21].

1) Kinematic state: the kinematic evolution model of sub-object *s* is defined as [17]

$$\begin{array}{l} x_{k}^{\left(i,s\right)}=(F_{k|k-1}^{\left(i,s\right)}⊗I_{d})x_{k-1}^{\left(i,s\right)}+w_{k}^{\left(i,s\right)}\\ w_{k}^{\left(i,s\right)}∼N(0,Q_{k|k-1}^{\left(i,s\right)}⊗X_{k}^{\left(i,s\right)}) \end{array}$$ (14)

where $F_{k|k-1}^{\left(i,s\right)}$ is kinematic state transition matrix, $w_{k}^{\left(i,s\right)}$ is zero mean Gaussian process noise with covariance $Q_{k|k-1}^{\left(i,s\right)}⊗X_{k}^{\left(i,s\right)}$, and $Q_{k|k-1}^{\left(i,s\right)}$ is given by

$$Q_{k|k-1}^{\left(i,s\right)}={\left(\Sigma_{k|k-1}^{\left(i,s\right)}\right)}^{2}(1-e^{-2T/\theta })diag([0\;0\;1])$$ (15)

where ${\left(\Sigma_{k|k-1}^{\left(i,s\right)}\right)}^{2}$ is the variance of acceleration noise, *T* is sampling time interval, and *θ* is the maneuver correlation time constant.

2) Extension state: the extension evolution model of sub-object *s* is given by [6]

$$p^{X}(X_{k}^{\left(i,s\right)}|X_{k-1}^{\left(i,s\right)})=W(X_{k}^{\left(i,s\right)};\delta_{k}^{\left(i,s\right)},A_{k}^{\left(i,s\right)}X_{k-1}^{\left(i,s\right)}{\left(A_{k}^{\left(i,s\right)}\right)}^{\text{T}})$$ (16)

where $\delta_{k}^{\left(i,s\right)}$, which is related to the uncertainty of the extension evolution, can describe the dependence of the extension on size over time. $A_{k}^{\left(i,s\right)}$ can describe the dependence of the extension on orientation (if $A_{k}^{\left(i,s\right)}$ is a rotation matrix), size (e.g., $A_{k}^{\left(i,s\right)}=\lambda I_{d}$), or shape (if $A_{k}^{\left(i,s\right)}$ is some other matrix).

3) Measurement rate: the evolution of $\gamma_{k}^{\left(i,s\right)}$ of sub-object *s* is modeled by using exponential forgetting with a factor *η*_*k*−1_ > 1 [21]

$$\alpha_{k|k-1}^{\left(i,s\right)}=\frac{\alpha_{k-1}^{\left(i,s\right)}}{\eta_{k-1}},\beta_{k|k-1}^{\left(i,s\right)}=\frac{\beta_{k-1}^{\left(i,s\right)}}{\eta_{k-1}}$$ (17)

Note that the prediction (17) corresponds to keeping the expected value constant while increasing the variance by multiplying with *η*_*k*−1_.

**Remark 1** (a) Sub-objects belonging to the same extended target share kinematic dynamics, which guarantees that all the sub-objects move together.

That is, all the sub-objects belonging to *i*th extended target have the same $F_{k|k-1}^{\left(i,s\right)}$ and $Q_{k|k-1}^{\left(i,s\right)}$.

(b) Sub-objects belonging to the same extended target may have different extension evolution models.

The interpretation is as follows. The extension evolution model of sub-object *s* of *i*th extended target can be represented by $\delta_{k}^{\left(i,s\right)}$ and $A_{k}^{\left(i,s\right)}$ of (16). Therefore, $\left(\delta_{k}^{\left(i,s\right)},A_{k}^{\left(i,s\right)}\right)$ of sub-object *s* may be different from $\left(\delta_{k}^{\left(i,t\right)},A_{k}^{\left(i,t\right)}\right)$ of sub-object *t*, *t* ≠ *s*. Also, from (11), we can see that $B_{k}^{\left(i,s\right)}\neq B_{k}^{\left(i,t\right)}$, *t* ≠ *s*.

(c) Sub-objects belonging to the same extended target should be initialized differently to distinguish one another, even if they have the same model.

## ET-PHD filter

For ETT problem, the prediction equation of ET-PHD filter [14] is given by

$$D_{k|k-1}(\xi_{k})=\int p_{S}(\xi_{k-1})p_{k|k-1}(\xi_{k}|\xi_{k-1})D_{k-1}(\xi_{k-1})d\xi_{k-1}+D_{k}^{b}(\xi_{k})$$ (18)

where target spawning problem is omitted, and

- *p*_*S*_(·) is the survival probability as a function of the target state;
- *p*_*k*_|_*k*−1_(·|·) is the state transition density;
- $D_{k}^{b}(\cdot )$ is the birth PHD.

The updating equation of ET-PHD filter is given by

$$D_{k|k}(\xi_{k}^{}|Z^{k})=L_{Z}(\xi_{k}^{})D_{k|k-1}(\xi_{k}^{}|Z^{k-1})$$ (19)

The pseudo-likelihood function *L*_**Z**_(***ξ***_*k*_) is given by

$$L_{Z}(\xi_{k})=1-(1-e^{-\gamma (\xi_{k})})p_{D}(\xi_{k})+e^{-\gamma (\xi_{k})}p_{D}(\xi_{k})\sum_{P∠Z_{k}}\omega_{p}\sum_{W\in P}\frac{\gamma {\left(\xi_{k}\right)}^{\left|W\right|}}{d_{W}}\prod_{z_{k}\in W}\frac{\phi_{z_{k}}(\xi_{k})}{\lambda_{k}c_{k}(z_{k})}$$ (20)

where

- $\lambda_{k}≜\beta_{FA,k}S$ is the mean number of clutter measurements;
- $c_{k}(z_{k})=1/S$ is the spatial distribution probability density of the clutter;
- $P∠Z_{k}$ denotes that P partitions the measurement set **Z**_*k*_ into non-empty subsets *W*. When used under a summation sign, the summation is over all possible partitions;
- W∈P denotes that *W* is a subset in the partition P. When used under a summation sign, the summation is over all subsets in the partition;
- *ω*_*p*_ and *d*_*W*_ are defined as follows
  $$\omega_{p}=\frac{\prod_{W\in P}d_{W}}{\sum_{P^{\prime }∠Z_{k}}\prod_{W^{\prime }\in P^{\prime }}d_{W^{\prime }}}$$ (21)
  $$d_{W}=\delta_{\left|W\right|,1}+D_{k|k-1}\left[p_{D}\gamma^{\left|W\right|}e^{-\gamma }\prod_{z_{k}\in W}\frac{\phi_{z_{k}}(\cdot )}{\lambda_{k}c_{k}(z_{k})}\right]$$ (22)

## Improved GGIW-PHD filter based on sub-random matrices for NETT

In this section, an improved GGIW-PHD filter based on sub-random matrices is presented.

### Assumptions

For the derivation of the prediction and updating equations, a number of assumptions are made.

Assumption 1: at time *k*, the PHD is denoted by an unnormalized mixture of GGIW distributions

$$D_{k|k}(\xi )=\sum_{i=1}^{J_{k|k}}\sum_{s=1}^{n_{k}^{\left(i,s\right)}}w_{k|k}^{\left(i,s\right)}GGIW(\xi_{k}^{\left(i,s\right)};\zeta_{k|k}^{\left(i,s\right)})$$ (23)

where *J*_*k*|*k*_ is the number of updated hypothesized tracks, and $w_{k|k}^{\left(i,s\right)}$ is the weight for sub-object *s* of *i*th hypothesized track.

Assumption 2: the birth PHD $D_{k}^{b}(\cdot )$ is an unnormalized mixture of GGIW distributions.

Assumption 3: the survival probability is state independent, i.e., *p*_*S*_(***ξ***_*k*_) = *p*_*S*_.

Assumption 4: the detection probability *p*_*D*_(·) satisfies, as shown in [23]

$$p_{D}(\xi_{k}^{\left(i,s\right)})GGIW(\xi_{k}^{\left(i,s\right)};\zeta_{k|k-1}^{\left(i,s\right)})\approx p_{D}(\zeta_{k|k-1}^{\left(i,s\right)})GGIW(\xi_{k}^{\left(i,s\right)};\zeta_{k|k-1}^{\left(i,s\right)})$$ (24)

### Prediction

Based on (13) and Assumptions 1 and 3, the prediction part of existing sub-objects of (18) can be converted to

$$D_{k|k-1}(\xi )=\sum_{i=1}^{J_{k-1}}\sum_{s=1}^{n_{k-1}^{\left(i,s\right)}}p_{S}w_{k-1}^{\left(i,s\right)}\underset{Measurement\;rate}{\underset{︸}{\int G(\gamma_{k-1}^{\left(i,s\right)};\alpha_{k-1}^{\left(i,s\right)},\beta_{k-1}^{\left(i,s\right)})p^{\gamma }(\gamma_{k}^{\left(i,s\right)}|\gamma_{k-1}^{\left(i,s\right)})d\gamma_{k-1}^{\left(i,s\right)}}}\times \underset{Kinematic\;part}{\underset{︸}{\int \begin{array}{c} N(x_{k-1}^{\left(i,s\right)};m_{k-1}^{\left(i,s\right)},P_{k-1}^{\left(i,s\right)}⊗X_{k}^{\left(i,s\right)})\\ \times p^{x}(x_{k}^{\left(i,s\right)}|x_{k-1}^{\left(i,s\right)}\text{,}X_{k}^{\left(i,s\right)}) \end{array}dx_{k-1}^{\left(i,s\right)}}}\cdot \underset{Extension\;part}{\underset{︸}{\int \begin{array}{c} IW_{d}\left(X_{k-1}^{\left(i,s\right)};v_{k-1}^{\left(i,s\right)},V_{k-1}^{\left(i,s\right)}\right)\\ \times p^{X}(X_{k}^{\left(i,s\right)}|X_{k-1}^{\left(i,s\right)}) \end{array}dX_{k-1}^{\left(i,s\right)}}}$$ (25)

Using the kinematic evolution model (14), the kinematic part of the prediction becomes

$$\int N(x_{k}^{\left(i,s\right)};(F_{k|k-1}^{\left(i,s\right)}⊗I_{d})x_{k-1}^{\left(i,s\right)},Q_{k|k-1}^{\left(i,s\right)}⊗X_{k}^{\left(i,s\right)})N(x_{k-1}^{\left(i,s\right)};m_{k-1}^{\left(i,s\right)},P_{k-1}^{\left(i,s\right)}⊗X_{k}^{\left(i,s\right)})dx_{k-1}^{\left(i,s\right)}=N(x_{k}^{\left(i,s\right)};m_{k|k-1}^{\left(i,s\right)},P_{k|k-1}^{\left(i,s\right)}⊗X_{k}^{\left(i,s\right)})$$ (26)

where

$$\begin{array}{c} m_{k|k-1}^{\left(i,s\right)}=(F_{k|k-1}^{\left(i,s\right)}⊗I_{d})m_{k-1}^{\left(i,s\right)}\\ P_{k|k-1}^{\left(i,s\right)}=F_{k|k-1}^{\left(i,s\right)}P_{k-1}^{\left(i,s\right)}{\left(F_{k|k-1}^{\left(i,s\right)}\right)}^{\text{T}}+Q_{k|k-1}^{\left(i,s\right)} \end{array}$$ (27)

The prediction of measurement rate has been given in (17). Also, using the extension evolution model (16), the extension part of the prediction becomes

$$\int W(X_{k}^{\left(i,s\right)};\delta_{k}^{\left(i,s\right)},A_{k}^{\left(i,s\right)}X_{k-1}^{\left(i,s\right)}{\left(A_{k}^{\left(i,s\right)}\right)}^{\text{T}})IW_{d}(X_{k-1}^{\left(i,s\right)};v_{k-1}^{\left(i,s\right)},V_{k-1}^{\left(i,s\right)})dX_{k-1}^{\left(i,s\right)}=GB_{d}^{\text{II}}(X_{k}^{\left(i,s\right)};a,b,A_{k}^{\left(i,s\right)}V_{k-1}^{\left(i,s\right)}{\left(A_{k}^{\left(i,s\right)}\right)}^{\text{T}},0)$$ (28)

where $GB_{d}^{\text{II}}(\cdot )$ is the generalized beta type II (GBII) distribution, $a=\delta_{k}^{\left(i,s\right)}/2$, and $b=(v_{k-1}^{\left(i,s\right)}-d-1)/2$. For detailed derivation of (28), see Appendix A of [6]. To achieve recursive estimation, the GBII distribution is approximated by an inverse Wishart distribution base on moment matching [6], then the distribution (28) is converted to

$$IW(X_{k}^{\left(i,s\right)}\text{;}v_{k|k-1}^{\left(i,s\right)},V_{k|k-1}^{\left(i,s\right)})$$ (29a)

with

$$v_{k|k-1}^{\left(i,s\right)}=\frac{\delta_{k}^{\left(i,s\right)}\text{(}\lambda_{k-1}^{\left(i,s\right)}+1)(\lambda_{k-1}^{\left(i,s\right)}-1)(\lambda_{k-1}^{\left(i,s\right)}-2)}{{\left(\lambda_{k-1}^{\left(i,s\right)}\right)}^{2}(\lambda_{k-1}^{\left(i,s\right)}\text{+}\delta_{k}^{\left(i,s\right)})}+2d+4$$ (29b)

$$V_{k|k-1}^{\left(i,s\right)}=\frac{\delta_{k}^{\left(i,s\right)}}{\lambda_{k-1}^{\left(i,s\right)}}(v_{k|k-1}^{\left(i,s\right)}-2d-2)A_{k}^{\left(i,s\right)}V_{k-1}^{\left(i,s\right)}{\left(A_{k}^{\left(i,s\right)}\right)}^{\text{T}}$$ (29c)

where $\lambda_{k-1}^{\left(i,s\right)}=v_{k-1}^{\left(i,s\right)}-2d-2$. It is obtained in [6] that a scalar measure of GBII distribution is given by

$$M_{k|k-1}^{\text{GBII}}=\frac{\delta_{k}^{\left(i,s\right)}(\delta_{k}^{\left(i,s\right)}+\lambda_{k-1}^{\left(i,s\right)})\sum_{i=1}^{d}c_{ii}^{2}}{\text{(}\lambda_{k-1}^{\left(i,s\right)}+1)(\lambda_{k-1}^{\left(i,s\right)}-1)(\lambda_{k-1}^{\left(i,s\right)}-2)}$$ (30)

which makes

$$v_{k|k-1}^{\left(i,s\right)}=\frac{2\delta_{k}^{\left(i,s\right)}\text{(}\lambda_{k-1}^{\left(i,s\right)}+1)(\lambda_{k-1}^{\left(i,s\right)}-1)(\lambda_{k-1}^{\left(i,s\right)}\text{-2)}}{{\left(\lambda_{k-1}^{\left(i,s\right)}\right)}^{2}(\lambda_{k-1}^{\left(i,s\right)}\text{+}\delta_{k}^{\left(i,s\right)})}+2d+4$$ (31)

However, after derivation we find that the value of $M_{k|k-1}^{\text{GBII}}$ is twice as much as that of (30), resulting in (29b).

Therefore, the prediction PHD of existing sub-objects is

$$\begin{array}{lll} D_{k|k-1}(\xi ) & =\overset{J_{k-1}}{\underset{i=1}{\sum }}\overset{n_{k-1}^{\left(i,s\right)}}{\underset{s=1}{\sum }}w_{k|k-1}^{\left(i,s\right)}G(\gamma_{k}^{\left(i,s\right)};\alpha_{k|k-1}^{\left(i,s\right)},\beta_{k|k-1}^{\left(i,s\right)}) & \\ & \;\times N(x_{k}^{\left(i,s\right)};m_{k|k-1}^{\left(i,s\right)},P_{k|k-1}^{\left(i,s\right)}⊗X_{k}^{\left(i,s\right)})IW(X_{k}^{\left(i,s\right)};v_{k|k-1}^{\left(i,s\right)},V_{k|k-1}^{\left(i,s\right)}) & 32(\text{a})\\ & =\overset{J_{k-1}}{\underset{i=1}{\sum }}\overset{n_{k-1}^{\left(i,s\right)}}{\underset{s=1}{\sum }}w_{k|k-1}^{\left(i,s\right)}GGIW(\xi_{k}^{\left(i,s\right)};\zeta_{k|k-1}^{\left(i,s\right)}) & 32(\text{b}) \end{array}$$

where $w_{k|k-1}^{\left(i,s\right)}=p_{S}w_{k-1}^{\left(i,s\right)}$.

In addition, the birth PHD is defined as

$$D_{k}^{b}(\xi )=\sum_{i=1}^{J_{b,k}}\sum_{s=1}^{n_{k}^{\left(i,s\right)}}w_{b,k}^{\left(i,s\right)}GGIW(\xi_{k}^{\left(i,s\right)};\zeta_{b,k}^{\left(i,s\right)})$$ (33)

The full prediction PHD is the sum of the prediction PHD of existing sub-objects (32) and the birth PHD (33). The number of predicted hypothesized tracks is

$$J_{k|k-1}^{}=J_{k-1}^{}+J_{b,k}$$ (34)

### Updating

Suppose that the prediction PHD is denoted by

$$D_{k|k-1}(\xi )=\sum_{i=1}^{J_{k|k-1}}\sum_{s=1}^{n_{k}^{\left(i,s\right)}}w_{k|k-1}^{\left(i,s\right)}GGIW(\xi_{k}^{\left(i,s\right)};\zeta_{k|k-1}^{\left(i,s\right)})$$ (35)

then the updating PHD is given by

$$D_{k|k}(\xi )=D_{k|k}^{ND}(\xi )+\sum_{P∠Z_{k}}\sum_{W\in P}D_{k|k}^{D}(\xi ,W)$$ (36)

where no detection part $D_{k|k}^{ND}(\xi )$ can be easily obtained according to (19), (20), and (35)

$$\begin{array}{c} D_{k|k}^{ND}(\xi )=\overset{J_{k|k-1}}{\underset{i=1}{\sum }}\overset{n_{k}^{\left(i,s\right)}}{\underset{s=1}{\sum }}(1-p_{D}^{\left(i,s\right)})w_{k|k-1}^{\left(i,s\right)}GGIW(\xi_{k}^{\left(i,s\right)};\zeta_{k|k-1}^{\left(i,s\right)})\\ +\overset{J_{k|k-1}}{\underset{i=1}{\sum }}\overset{n_{k}^{\left(i,s\right)}}{\underset{s=1}{\sum }}p_{D}^{\left(i,s\right)}w_{k|k-1}^{\left(i,s\right)}{\left(\frac{\beta_{k|k-1}^{\left(i,s\right)}}{\beta_{k|k-1}^{\left(i,s\right)}+1}\right)}^{\alpha_{k|k-1}^{\left(i,s\right)}}GGIW(\xi_{k}^{\left(i,s\right)};{\bar{\zeta }}_{k|k-1}^{\left(i,s\right)}) \end{array}$$ (37)

where the parameters in ${\bar{\zeta }}_{k|k-1}^{\left(i,s\right)}$ are the same as that in $\zeta_{k|k-1}^{\left(i,s\right)}$ except that ${\bar{\beta }}_{k|k-1}^{\left(i,s\right)}=\beta_{k|k-1}^{\left(i,s\right)}+1$.

The calculation of detection part $D_{k|k}^{D}(\xi ,W)$ requires the product of

$$e^{-\gamma_{k}^{\left(i,s\right)}}{\left(\gamma_{k}^{\left(i,s\right)}\right)}^{\left|W\right|}\prod_{z_{k}\in W}\frac{\phi_{z_{k}}(\xi_{k}^{\left(i,s\right)})}{\lambda_{k}c_{k}(z_{k})}=e^{-\gamma_{k}^{\left(i,s\right)}}{\left(\gamma_{k}^{\left(i,s\right)}\right)}^{\left|W\right|}\beta_{FA,k}^{-|W|}\prod_{z_{k}\in W}\phi_{z_{k}}(\xi_{k}^{\left(i,s\right)})$$ (38)

and the prediction component $GGIW(\xi_{k}^{\left(i,s\right)};\zeta_{k|k-1}^{\left(i,s\right)})$. The product can be rewritten as

$$\begin{array}{l} \beta_{FA,k}^{-|W|}L_{k}^{\left(i,s,W\right)}GGIW(\xi_{k}^{\left(i,s,W\right)};\zeta_{k}^{\left(i,s,W\right)})\\ =\beta_{FA,k}^{-|W|}L_{k}^{\left(i,s,W\right)}G(\gamma_{k}^{\left(i,s,W\right)};\alpha_{k|k}^{\left(i,s,W\right)},\beta_{k|k}^{\left(i,s,W\right)})N(x_{k}^{\left(i,s,W\right)};m_{k|k}^{\left(i,s,W\right)},P_{k|k}^{\left(i,s,W\right)}⊗X_{k}^{\left(i,s,W\right)})\\ \times IW(X_{k}^{\left(i,s,W\right)};v_{k|k}^{\left(i,s,W\right)},V_{k|k}^{\left(i,s,W\right)}) \end{array}$$ (39)

the details of the derivation are given in S1 File. The updating parameters in (39) are given by

$$\alpha_{k|k}^{\left(i,s,W\right)}=\alpha_{k|k-1}^{\left(i,s\right)}+\left|W\right|$$ (40a)

$$\beta_{k|k}^{\left(i,s,W\right)}=\beta_{k|k-1}^{\left(i,s\right)}+1$$ (40b)

$$m_{k|k}^{\left(i,s,W\right)}=m_{k|k-1}^{\left(i,s\right)}+(K_{k|k-1}^{\left(i,s,W\right)}⊗I_{d})\varepsilon_{k|k-1}^{\left(i,s,W\right)}$$ (40c)

$$P_{k|k}^{\left(i,s,W\right)}=P_{k|k-1}^{\left(i,s\right)}-K_{k|k-1}^{\left(i,s,W\right)}S_{k|k-1}^{\left(i,s,W\right)}{\left(K_{k|k-1}^{\left(i,s,W\right)}\right)}^{\text{T}}$$ (40d)

$$v_{k|k}^{\left(i,s,W\right)}=v_{k|k-1}^{\left(i,s\right)}+\left|W\right|$$ (40e)

$$V_{k|k}^{\left(i,s,W\right)}=V_{k|k-1}^{\left(i,s\right)}+N_{k|k-1}^{\left(i,s,W\right)}+{\left(B_{k}^{\left(i,s\right)}\right)}^{-1}Z_{k}^{W}{\left(B_{k}^{\left(i,s\right)}\right)}^{-\text{T}}$$ (40f)

where

$${\bar{z}}_{k}^{W}=(1/\left|W\right|)\sum_{z_{k}^{\left(j\right)}\in W}z_{k}^{\left(j\right)}$$ (41a)

$$Z_{k}^{W}=\sum_{z_{k}^{\left(j\right)}\in W}(z_{k}^{\left(j\right)}-{\bar{z}}_{k}^{W}){\left(z_{k}^{\left(j\right)}-{\bar{z}}_{k}^{W}\right)}^{\text{T}}$$ (41b)

$$S_{k|k-1}^{\left(i,s,W\right)}=H_{k}^{s}P_{k|k-1}^{\left(i,s\right)}{\left(H_{k}^{s}\right)}^{\text{T}}+(1/\left|W\right|){\left|B_{k}^{\left(i,s\right)}\right|}^{2/d}$$ (41c)

$$K_{k|k-1}^{\left(i,s,W\right)}=P_{k|k-1}^{\left(i,s\right)}{\left(H_{k}^{s}\right)}^{\text{T}}{\left(S_{k|k-1}^{\left(i,s,W\right)}\right)}^{-1}$$ (41d)

$$\varepsilon_{k|k-1}^{\left(i,s,W\right)}={\bar{z}}_{k}^{W}-(H_{k}^{s}⊗I_{d})m_{k|k-1}^{\left(i,s\right)}$$ (41e)

$$N_{k|k-1}^{\left(i,s,W\right)}={\left(S_{k|k-1}^{\left(i,s,W\right)}\right)}^{-1}\varepsilon_{k|k-1}^{\left(i,s,W\right)}{\left(\varepsilon_{k|k-1}^{\left(i,s,W\right)}\right)}^{\text{T}}$$ (41f)

The likelihood function is given by

$$\begin{array}{l} {ℒ}_{k}^{\left(i,s,W\right)}={\left(\pi^{\left|W\right|}\left|W\right|S_{k|k-1}^{\left(i,s,W\right)}\right)}^{{}^{-d/2}}{\left|B_{k}^{\left(i,s\right)}\right|}^{-(\left|W\right|-1)}\times \frac{\beta_{k|k-1}^{\left(i,s\right)}{}^{\alpha_{k|k-1}^{\left(i,s\right)}}}{\beta_{k|k}^{\left(i,s,W\right)}{}^{\alpha_{k|k}^{\left(i,s,W\right)}}}\frac{\Gamma (\alpha_{k|k}^{\left(i,s,W\right)})}{\Gamma (\alpha_{k|k-1}^{\left(i,s\right)})}\\ \;\;\;\;\;\;\;\;\;\;\;\;\;\times \frac{{\left|V_{k|k-1}^{\left(i,s\right)}\right|}^{(v_{k|k-1}^{\left(i,s\right)}-d-1)/2}}{{\left|V_{k|k}^{\left(i,s,W\right)}\right|}^{(v_{k|k}^{\left(i,s,W\right)}-d-1)/2}}\frac{\Gamma_{d}\left((v_{k|k}^{\left(i,s,W\right)}-d-1)/2\right)}{\Gamma_{d}\left((v_{k|k-1}^{\left(i,s\right)}-d-1)/2\right)} \end{array}$$ (41g)

According to (22) and (39), *d*_*W*_ can be easily obtained

$$d_{W}=\delta_{\left|W\right|,1}+\sum_{i=1}^{J_{k|k-1}}\sum_{s=1}^{n_{k}^{\left(i,s\right)}}w_{k|k-1}^{\left(i,s\right)}p_{D}^{\left(i,s\right)}\beta_{FA,k}^{-|W|}L_{k}^{\left(i,s,W\right)}$$ (42)

Then the updating weight is given by

$$w_{k|k}^{\left(i,s,W\right)}=\frac{\omega_{p}}{d_{W}}w_{k|k-1}^{\left(i,s\right)}p_{D}^{\left(i,s\right)}\beta_{FA,k}^{-|W|}L_{k}^{\left(i,s,W\right)}$$ (43)

where *ω*_*p*_ can be calculated by (21). The full updating PHD is the sum of no detection part PHD (37) and detection part PHD that is of the form in (23) with weights (43) and parameters (40).

Let $\left|P_{p}\right|$ denotes the number of subset *W* in the *p*th partition, and *P* denotes the number of partitions. Then the number of updated hypothesized tracks is

$$J_{k|k}=2J_{k|k-1}+J_{k|k-1}\sum_{p=1}^{P}\left|P_{p}\right|$$ (44)

### Pruning and merging

From the prediction and updating processes, we can see that the number of GGIW components increases rapidly, which leads to a huge computational complexity. To prevent the unbounded growth of components, pruning and merging are indispensable. Firstly, the components, whose weights fall below a predetermined threshold *T* or measurement rate estimates are less than 1, are pruned. The merging methods for GGIW components have been discussed in [21,29], where the merging criterions for different state variables (e.g., *γ*_*k*_ and ***x***_*k*_) are individually obtained by Kullback-Leibler divergence, which, however, is boundless. Here, an integrative merging criterion is obtained by using Hellinger distance that is widely used to quantify the similarity between two probability distributions. Also, the distance ranges from 0 to 1. The Hellinger distance *d*_*μv*_ for GGIW component pair (*μ*,*v*) is given by

$$d_{\mu \nu }=\sqrt{1-d_{\mu \nu }^{\gamma }d_{\mu \nu }^{x}}$$ (45a)

$$d_{\mu \nu }^{\gamma }=\frac{\Gamma ({\hat{\alpha }}_{k|k}^{\left(\mu ,\nu \right)})}{\sqrt{\Gamma (\alpha_{k|k}^{\left(\mu \right)})\Gamma (\alpha_{k|k}^{\left(\nu \right)})}}\frac{\sqrt{\beta_{k|k}^{\left(\mu \right)}{}^{\alpha_{k|k}^{\left(\mu \right)}}\beta_{k|k}^{\left(\nu \right)}{}^{\alpha_{k|k}^{\left(\nu \right)}}}}{{\hat{\beta }}_{k|k}^{\left(\mu ,\nu \right)}{}^{{\hat{\alpha }}_{k|k}^{\left(\mu ,\nu \right)}}}$$ (45b)

$$d_{\mu \nu }^{x}=\text{det}{\left({\hat{P}}_{k|k}^{\left(\mu \right)}\times {\hat{P}}_{k|k}^{\left(\nu \right)}\right)}^{1/4}/\text{det}{\left({\hat{P}}_{k|k}^{\left(\mu ,\nu \right)}\right)}^{1/2}\text{exp}\left\{-\frac{1}{8}{\left({\hat{m}}_{k|k}^{\left(\mu ,\nu \right)}\right)}^{\text{T}}{\left({\hat{P}}_{k|k}^{\left(\mu ,\nu \right)}\right)}^{-1}({\hat{m}}_{k|k}^{\left(\mu ,\nu \right)})\right\}$$ (45c)

where ${\hat{P}}_{k|k}^{\left(\mu \right)}$ and ${\hat{P}}_{k|k}^{\left(\nu \right)}$ can be obtained by (7), and

$${\hat{\alpha }}_{k|k}^{\left(\mu ,\nu \right)}=(\alpha_{k|k}^{\left(\mu \right)}+\alpha_{k|k}^{\left(\nu \right)})/2$$ (46a)

$${\hat{\beta }}_{k|k}^{\left(\mu ,\nu \right)}=(\beta_{k|k}^{\left(\mu \right)}+\beta_{k|k}^{\left(\nu \right)})/2$$ (46b)

$${\hat{m}}_{k|k}^{\left(\mu ,\nu \right)}=m_{k|k}^{\left(\mu \right)}-m_{k|k}^{\left(\nu \right)}$$ (46c)

$${\hat{P}}_{k|k}^{\left(\mu ,\nu \right)}=({\hat{P}}_{k|k}^{\left(\mu \right)}+{\hat{P}}_{k|k}^{\left(\nu \right)})/2$$ (46d)

Instead of using the weighted mean of parameters as the merging result, we propose a novel merging scheme that is a generalized version of the moment matching method given in [6]. The details of the derivation are given in S2 File, and the pruning and merging schemes are given in Table 1.

**Table 1:** Pseudocode for GGIW-PHD filter pruning and merging scheme.

1: **input**: A truncation threshold *T*, a merging threshold *U*, updating GGIW components ${\left\{w_{k|k}^{\left(\nu \right)},\alpha_{k|k}^{\left(\nu \right)},\beta_{k|k}^{\left(\nu \right)}\text{,}m_{k|k}^{\left(\nu \right)},P_{k|k}^{\left(\nu \right)},v_{k|k}^{\left(\nu \right)},V_{k|k}^{\left(\nu \right)}\right\}}_{\nu =1}^{J_{k|k}^{}}$, and a maximum allowable number of GGIW components *J*_max_. 2: **initialize**: Set *l* = 0 and $I=\{\mu =1,\ldots ,J_{k|k}|w_{k|k}^{\left(\mu \right)}>T\;\&\;\alpha_{k|k}^{\left(\mu \right)}/\beta_{k|k}^{\left(\mu \right)}\geq 1\}$. 3: **repeat**: 4: *l = l + 1* 5: $\nu =\underset{\mu \in I}{\text{arg}\;\text{max}}w_{k|k}^{\left(\mu \right)}$ 6: *L* = {*μ* ∈ *I* | *d*_*μv*_ ≤ *U*}, 0 < d_*μv*_ < 1 7: ${\bar{w}}_{k|k}^{\left(l\right)}=\sum_{\mu \in L}w_{k|k}^{\left(\mu \right)}$ 8: Let ${\bar{w}}_{k|k}^{\left(l\right)}$ and ${\left\{w_{k|k}^{\left(\mu \right)},\alpha_{k|k}^{\left(\mu \right)},\beta_{k|k}^{\left(\mu \right)}\text{,}m_{k|k}^{\left(\mu \right)},P_{k|k}^{\left(\mu \right)},v_{k|k}^{\left(\mu \right)},V_{k|k}^{\left(\mu \right)}\right\}}_{\mu \in L}$ as the input of the merging method in Table B1 given in S2 File, and let the output $\left\{{\bar{\alpha }}_{k|k}^{\left(l\right)},{\bar{\beta }}_{k|k}^{\left(l\right)},{\bar{m}}_{k|k}^{\left(l\right)},{\bar{P}}_{k|k}^{\left(l\right)},{\bar{v}}_{k|k}^{\left(l\right)},{\bar{V}}_{k|k}^{\left(l\right)}\right\}$ as the merging GGIW parameters. 9: *I = I\L* 10: **until**: *I* = ∅ 11: If *l* > *J*_max_ then replace ${\left\{{\bar{w}}_{k|k}^{\left(\nu \right)},{\bar{\alpha }}_{k|k}^{\left(\nu \right)},{\bar{\beta }}_{k|k}^{\left(\nu \right)},{\bar{m}}_{k|k}^{\left(\nu \right)},{\bar{P}}_{k|k}^{\left(\nu \right)},{\bar{v}}_{k|k}^{\left(\nu \right)},{\bar{V}}_{k|k}^{\left(\nu \right)}\right\}}_{\nu =1}^{l}$ by those of the *J*_max_ GGIW components with largest weights. 12: **output:${\left\{{\bar{w}}_{k|k}^{\left(\nu \right)},{\bar{\alpha }}_{k|k}^{\left(\nu \right)},{\bar{\beta }}_{k|k}^{\left(\nu \right)},{\bar{m}}_{k|k}^{\left(\nu \right)},{\bar{P}}_{k|k}^{\left(\nu \right)},{\bar{v}}_{k|k}^{\left(\nu \right)},{\bar{V}}_{k|k}^{\left(\nu \right)}\right\}}_{\nu =1}^{l}$**.

## MM-GGIW-PHD filter based on sub-random matrices for MNETT

The proposed GGIW-PHD filter in Section 5 is effective for NETT in non-maneuvering scenes. For MNETT, directly applying this approach may result in unacceptable estimates. When the extended targets maneuver, the non-maneuver models characterizing the sub-objects are not in accordance with the actuality, which may lead to overall extension estimation deviations [27]. This is proved later by simulation. To handle this model mismatch problem, an MM approach based on model combination is proposed for a single-target scene in [27]. In this section, the model combination method is incorporated into MM-PHD filter [30] to track multiple maneuvering NE extended targets.

### Model combination

Firstly, we define a model set to describe the overall maneuver processes of the extended targets

$${\bar{M}}_{k}=\{{\bar{m}}_{k}^{r},r=1,\ldots ,N_{k}\}$$ (47)

where *N*_*k*_ is the number of the models. It is assumed that measurements depend on the models only through the kinematic and extension states. Thus, only the kinematic and extension evolution models of the extended targets are modeled here. ${\bar{m}}_{k}^{r}$ is defined as

$$\{\begin{array}{l} x_{k}^{i}=({\bar{F}}_{k|k-1}^{r}⊗I_{d})x_{k-1}^{i}+{\bar{w}}_{k}^{i,r}\\ {\bar{w}}_{k}^{i,r}∼N(0,{\bar{Q}}_{k|k-1}^{r}⊗X_{k}^{i})\\ p^{X}(X_{k}^{i}|X_{k-1}^{i})=W(X_{k}^{i};{\bar{\delta }}_{k}^{r},{\bar{A}}_{k}^{r}X_{k-1}^{i}{\left({\bar{A}}_{k}^{r}\right)}^{\text{T}}) \end{array}$$ (48)

where $x_{k}^{i}$ and $X_{k}^{i}$ denote the kinematic and extension states of *i*th extended target, and

$${\bar{Q}}_{k|k-1}^{r}={\left({\bar{\Sigma }}_{k|k-1}^{r}\right)}^{2}(1-e^{-2T/\theta })diag([0\;0\;1])$$ (49)

As presented in Section 3.4, the sub-object *s* of *i*th extended target has the model $m_{k}^{\left(i,s\right)}$ with the form of (48). Actually, the overall evolution of an extended target affects the individual evolutions of its sub-objects. That is, when the overall model ${\bar{m}}_{k}^{r}$ is in effect, the sub-object *s* of *i*th extended target will be characterized by a new model $m_{k}^{\left(i,s|r\right)}$ that is the combination of ${\bar{m}}_{k}^{r}$ and $m_{k}^{\left(i,s\right)}$.

${\bar{m}}_{k}^{r}$ and $m_{k}^{\left(i,s\right)}$ have the following properties:

1) All the sub-objects belonging to the same extended target share kinematic dynamics characterized by $F_{k|k-1}^{\left(i,s\right)}$ and $\Sigma_{k|k-1}^{\left(i,s\right)}$ but have different extension evolutions characterized by $\delta_{k}^{\left(i,s\right)}$ and $A_{k}^{\left(i,s\right)}$. (see **Remark 1**)

2) Models $m_{k}^{\left(i,s\right)}$ and ${\bar{m}}_{k}^{r}$ have the same kinematic state transition matrix, i.e.,$F_{k|k-1}^{\left(i,s\right)}={\bar{F}}_{k|k-1}^{r}$, ∀*i*∈{1, …, *N*_*x*,*k*_},$s\in \{1,\ldots ,n_{k}^{\left(i,s\right)}\}$, *r*∈{1, …, *N*_*k*_}.

3) ${\bar{m}}_{k}^{r}$ is characterized by $\left\{{\bar{\Sigma }}_{k|k-1}^{r},{\bar{\delta }}_{k}^{r},{\bar{A}}_{k}^{r}\right\}$, which is valid because using these parameters is enough for maneuvering extended target tracking.

So, the determination of $m_{k}^{\left(i,s|r\right)}$ reduces to obtaining parameters $\left\{\Sigma_{k|k-1}^{\left(i,s|r\right)},\delta_{k}^{\left(i,s|r\right)},A_{k}^{\left(i,s|r\right)}\right\}$ given $\left\{{\bar{\Sigma }}_{k|k-1}^{r},{\bar{\delta }}_{k}^{r},{\bar{A}}_{k}^{r}\right\}$ of ${\bar{m}}_{k}^{r}$ and $\left\{\Sigma_{k|k-1}^{\left(i,s\right)},\delta_{k}^{\left(i,s\right)},A_{k}^{\left(i,s\right)}\right\}$ of $m_{k}^{\left(i,s\right)}$. The model combination method is given by

$$\{\begin{array}{l} \Sigma_{k|k-1}^{\left(i,s|r\right)}=\Sigma_{k|k-1}^{\left(i,s\right)}{\bar{\Sigma }}_{k|k-1}^{r}/{\bar{\Sigma }}_{k|k-1}^{1}\\ \delta_{k}^{\left(i,s|r\right)}=\delta_{k}^{\left(i,s\right)}{\bar{\delta }}_{k}^{r}/{\bar{\delta }}_{k}^{1}\\ A_{k}^{\left(i,s|r\right)}={\bar{A}}_{k}^{r}{\left({\bar{A}}_{k}^{1}\right)}^{-1}A_{k}^{\left(i,s\right)} \end{array}$$ (50)

The interpretation is given as follows.

1) If the overall non-maneuver model ${\bar{m}}_{k}^{1}(r=1)$ is in effect or the extended target is not maneuvering, $m_{k}^{\left(i,s|r\right)}$ reduces to $m_{k}^{\left(i,s\right)}$.

2) $\Sigma_{k|k-1}^{\left(i,s|r\right)}$ increases the uncertainty of the kinematic evolution because ${\bar{\Sigma }}_{k|k-1}^{r}>{\bar{\Sigma }}_{k|k-1}^{1}$ during maneuver.

3) $A_{k}^{\left(i,s|r\right)}$ denotes that the sub-object *s* performs an individual rotation $A_{k}^{\left(i,s\right)}$ on top of an overall rotation ${\bar{A}}_{k}^{r}$(${\bar{A}}_{k}^{1}=\lambda I_{d}$) when *i*th extended target is maneuvering.

4) $\delta_{k}^{\left(i,s|r\right)}$ increases the uncertainty of extension evolution because ${\bar{\delta }}_{k}^{r}<{\bar{\delta }}_{k}^{1}$ during maneuver.

### Assumptions

To derive the MM-GGIW-PHD filter, a number of assumptions are made here.

Assumption 5: the dynamic evolution model of sub-object *s* of *i*th extended target is given by

$$p(\xi_{k}^{\left(i,s\right)},m_{k}^{\left(i,s|r\right)}|\xi_{k-1}^{\left(i,s\right)},m_{k-1}^{\left(i,s|r^{\prime }\right)})=p(\xi_{k}^{\left(i,s\right)}|\xi_{k-1}^{\left(i,s\right)},m_{k}^{\left(i,s|r\right)})t_{k|k-1}(r|r^{\prime })$$ (51)

where $p(\xi_{k}^{\left(i,s\right)}|\xi_{k-1}^{\left(i,s\right)},m_{k}^{\left(i,s|r\right)})$ satisfies the form of (13) based on $m_{k}^{\left(i,s|r\right)}$, and $t_{k|k-1}(r|r^{\prime })$ is the model transition probability from $m_{k-1}^{\left(i,s|r^{\prime }\right)}$ to $m_{k}^{\left(i,s|r\right)}$.

Assumption 6: the birth PHD is an unnormalized mixture of GGIW distributions

$$D_{k}^{b}(\xi ,{\bar{m}}_{k}^{r})=\pi_{k}^{r}\sum_{i=1}^{J_{b,k|r}}\sum_{s=1}^{n_{k}^{\left(i,s\right)}}w_{b,k}^{\left(i,s|r\right)}GGIW(\xi_{k}^{\left(i,s\right)};\zeta_{b,k}^{\left(i,s|r\right)})$$ (52)

where $\pi_{k}^{r}$ is the birth intensity of model ${\bar{m}}_{k}^{r}$.

### MM-GGIW-PHD filter

1) Prediction: assuming that at time *k* − 1, the posterior PHD D_*k*−1_(·) has the form similar to (23)

$$D_{k-1}(\xi ,{\bar{m}}_{k-1}^{r^{\prime }})=\sum_{i=1}^{J_{k-1|r^{\prime }}}\sum_{s=1}^{n_{k-1}^{\left(i,s\right)}}w_{k-1}^{\left(i,s|r^{\prime }\right)}GGIW(\xi_{k-1}^{\left(i,s\right)};\zeta_{k-1}^{\left(i,s|r^{\prime }\right)})$$ (53)

where *r*′ ∈ {1, …, *N*_*k*−1_}. The prediction of existing sub-objects for model ${\bar{m}}_{k}^{r}$ is given by

$$D_{k|k-1}(\xi ,{\bar{m}}_{k}^{r})=\sum_{r^{\prime }}\sum_{i=1}^{J_{k-1|r^{\prime }}}\sum_{s=1}^{n_{k-1}^{\left(i,s\right)}}w_{k|k-1}^{\left(i,s|r,r^{\prime }\right)}\int p(\xi_{k}^{\left(i,s\right)}|\xi_{k-1}^{\left(i,s\right)},m_{k}^{\left(i,s|r\right)})GGIW(\xi_{k-1}^{\left(i,s\right)};\zeta_{k-1}^{\left(i,s|r^{\prime }\right)})d\xi_{k-1}^{\left(i,s\right)}$$ (54)

where $w_{k|k-1}^{\left(i,s|r,r^{\prime }\right)}=p_{S}(m_{k-1}^{\left(i,s|r^{\prime }\right)})t_{k|k-1}(r|r^{\prime })w_{k-1}^{\left(i,s|r^{\prime }\right)}$. The integral term of (54) can be calculated easily according to (25).

The full prediction PHD is the sum of the prediction PHD of existing sub-objects (54) and the birth PHD (52). For model ${\bar{m}}_{k}^{r}$, the number of predicted hypothesized tracks is

$$J_{k|k-1,r}=\sum_{r^{\prime }=1}^{N_{k-1}}J_{k-1|r^{\prime }}+J_{b,k|r}$$ (55)

2) Updating: assuming that the prediction PHD has the form

$$D_{k|k-1}(\xi ,{\bar{m}}_{k}^{r})=\sum_{i=1}^{J_{k|k-1,r}}\sum_{s=1}^{n_{k}^{\left(i,s\right)}}w_{k|k-1}^{\left(i,s|r\right)}GGIW(\xi_{k}^{\left(i,s\right)};\zeta_{k|k-1}^{\left(i,s|r,r^{\prime }\right)})$$ (56)

The updating PHD can be easily obtained as in Section 5.3. For space consideration, the updating process is not repeated here. For model ${\bar{m}}_{k}^{r}$, the number of updated hypothesized tracks is

$$J_{k|k,r}^{}=2J_{k|k-1,r}+J_{k|k-1,r}\sum_{p=1}^{P_{r}}\left|P_{p,r}\right|$$ (57)

From the above derivation, we can find that the MM-GGIW-PHD filter requires *N*_*k*_ (the number of the models) PHD filters to operate in parallel, and the component number of each filter increases more rapidly than that of single model GGIW-PHD filter in Section 5. Thus, pruning and merging schemes given in Table 1 are also indispensable to prevent the unbounded growth of components for each PHD filter.

A feasible merging method for the extracted states of PHD filters corresponding to each overall model is presented next. Assuming that the extracted states of *N*_*k*_ PHD filters are denoted by

$$\left\{(\zeta_{k|k}^{\left(i,s|r\right)},{\bar{m}}_{k}^{r})|i=1,\ldots ,J_{k}^{r},s=1,\ldots ,n_{k}^{\left(i,s\right)},r=1,\ldots ,N_{k}\right\}$$ (58)

The merging method is similar to that in Table 1, but the difference is that the corresponding likelihoods (41g) of each extracted state are used as their weights, and the weighted mean of the states is regarded as the merging result.

## Measurement set partitioning

The proposed both GGIW-PHD filter and MM-GGIW-PHD filter require all partitions P of the current measurement set **Z**_*k*_. A partition is a division of **Z**_*k*_ into non-empty subsets *W*. Each subset *W* can be interpreted as containing measurements that all stem from the same source, either a sub-object or a clutter source.

The updating PHD (36) requires a summation over all possible partitions, which will quickly become computationally intractable because the number of partitions increases very rapidly as the number of measurements increases [3,4,20]. Several partitioning methods use a subset of partitions to approximate all possible partitions. Distance partition [3] is based on the fundamental insight that measurements that are all from the same extended target are spatially close to each other, and its first order improved version with the subpartition algorithm is presented to deal with the case of spatially close targets. However, for NETT problem, we intend to place measurements that all stem from the same sub-object into a subset *W*. Because the measurements of all the sub-objects belonging to the same extended target or of multiple spatially close targets are interwoven, distance partition is insufficient for the measurement set partitioning problem in this paper. Prediction partition [20,28] is a feasible method for this partitioning problem, however, it puts the measurement that falls into two or more prediction gates into the subset *W* corresponding to the prediction component with highest weight, which is highly possible to discard the correct partitions. Lan [27] takes this drawback into consideration, but no specific method is presented to get all feasible partitions. In addition, pseudo-likelihood method [27] is effective to control the increase of the number of partitions. In this section, a specific implementation of prediction partition with pseudo-likelihood method is presented. The detailed steps are given in Table 2.

**Table 2:** Pseudocode for prediction partition with pseudo-likelihood method.

1: **Input**: Measurement set $Z_{k}={\left\{z_{k}^{\left(j\right)}\right\}}_{j=1}^{N_{z,k}}$ and all prediction GGIW components ${\left\{w_{k|k-1}^{\left(i\right)},\xi_{k|k-1}^{\left(i\right)}\right\}}_{i=1}^{J_{k|k-1}}$ at time *k*. 2: **Initialize**: Set *l* = 0, $I=\{i=1,\ldots ,J_{k|k-1}|w_{k|k-1}^{\left(i\right)}>0.5\}$, *J* = {*J* = 1, …, *N*_***z***_,_*k*_}, $L_{j,j\in J}=\{i\in I|\xi_{k|k-1}^{\left(i\right)}\;\text{and}\;z_{k}^{\left(j\right)}\;\text{fulfill}\;(59)\}$, $M_{i,i\in I}=\{j\in J|{\text{z}}_{k}^{\left(j\right)}\;\text{and}\;\xi_{k|k-1}^{\left(i\right)}\;\text{fulfill}\;(59)\}$, $\tilde{J}=\{j\in J|\;|L_{j}|=1\}$, $\bar{J}=\{j\in J|\;|L_{j}|>1\}$, $\bar{I}=\{i\in I|M_{i}\cap \bar{J}\neq \emptyset \;\&M_{i}\cap \tilde{J}=\emptyset \}$, $\tilde{I}=\{i\in I|M_{i}\cap \tilde{J}\neq \emptyset \}$. 3: **Repeat**: For $j\in \bar{J}$ 4: **If** $L_{j}\subset \tilde{I}$, let *l* = *l* + 1, *F*_*l*_ = {*j*},*G*_*l*_ = *L*_*j*_,$\bar{J}=\bar{J}\backslash \{j\}$, **End**. 5: **If $L_{j}\cap \bar{I}\neq \emptyset$**, let *l* = *l* + 1, *A* = {*j*}, *B* = ∅, 6: **for** *i*_1_ = 1:size(*A*) 7: pre_size_B = size(*B*),$B=B\cup (L_{A(i_{1})}\cap \bar{I})$ 8: **for** *i*_2_ = (pre_size_B+1):size(*B*) 9: $A=A\cup M_{B(i_{2})}$ 10: **end** 11: **end** 12: Let *F*_*l*_ = *A* = {*j*_1_, …, *j*_*n*_}, *G*_*l*_ = ∅, 13: **for** $i_{1}=1:\text{size}(L_{j_{1}})$ … 14: **for** $i_{n}=1:\text{size}(L_{j_{n}})$ 15: **if** $B\subset \{L_{j_{1}}(i_{1}),\ldots ,L_{j_{n}}(i_{n})\}$ 16: $G_{l}=G_{l}\cup \{(L_{j_{1}}(i_{1}),\ldots ,L_{j_{n}}(i_{n}))\}$ 17: **end** 18: **end** 19: **end** 20:$\bar{J}=\bar{J}\backslash A$ 21: **End** 22: **Until: $\bar{J}=\emptyset$** 23: Obtain all partitions ${\left\{P_{p}\right\}}_{p=1}^{N}$, *N* = |*G*_1_| × |*G*_2_| ×⋯× |*G*_*l*_|. 24: If *N* > *η* then replace ${\left\{P_{p}\right\}}_{p=1}^{N}$ by those of the *η* partitions with largest likelihoods. 25: **Output:${\left\{P_{p}\right\}}_{p=1}^{N}$**.

*A*(*i*) denotes *i*th element in the set *A*.

Prediction partition uses the prediction GGIW components to validate the measurements for each sub-object. For components with $w_{k|k-1}^{\left(i,s\right)}>0.5$, the prediction gates for each sub-object can be defined as

$$G_{k}^{\left(z,i,s\right)}=\left\{\begin{array}{c} z:{\left(z_{k}^{\left(j\right)}-{\hat{z}}_{k|k-1}^{\left(i,s\right)}\right)}^{\text{T}}{\left(S_{k|k-1}^{\left(i,s,j\right)}V_{k|k-1}^{\left(i,s\right)}\right)}^{-1}\\ \cdot (z_{k}^{\left(j\right)}-{\hat{z}}_{k|k-1}^{\left(i,s\right)})\leq \Delta_{d}^{\left(i,s\right)} \end{array}\right\}$$ (59)

where ${\hat{z}}_{k|k-1}^{\left(i,s\right)}=(H_{k}^{s}⊗I_{d}){\text{m}}_{k|k-1}^{\left(i,s\right)}$, and $m_{k|k-1}^{\left(i,s\right)}$,$S_{k|k-1}^{\left(i,s,j\right)}$, and $V_{k|k-1}^{\left(i,s\right)}$ are respectively calculated by (27), (41c), and (29c) with $W=\{z_{k}^{\left(j\right)}\}$. The $\Delta_{d}^{\left(i,s\right)}$ is determined by the gate probability *P*_*G*_ as [27]

$$\Delta_{d}^{\left(i,s\right)}={\left(1-P_{G}\right)}^{-2/(v_{k|k-1}^{\left(i,s\right)}-d-2)}-1$$ (60)

**Fig 3:**
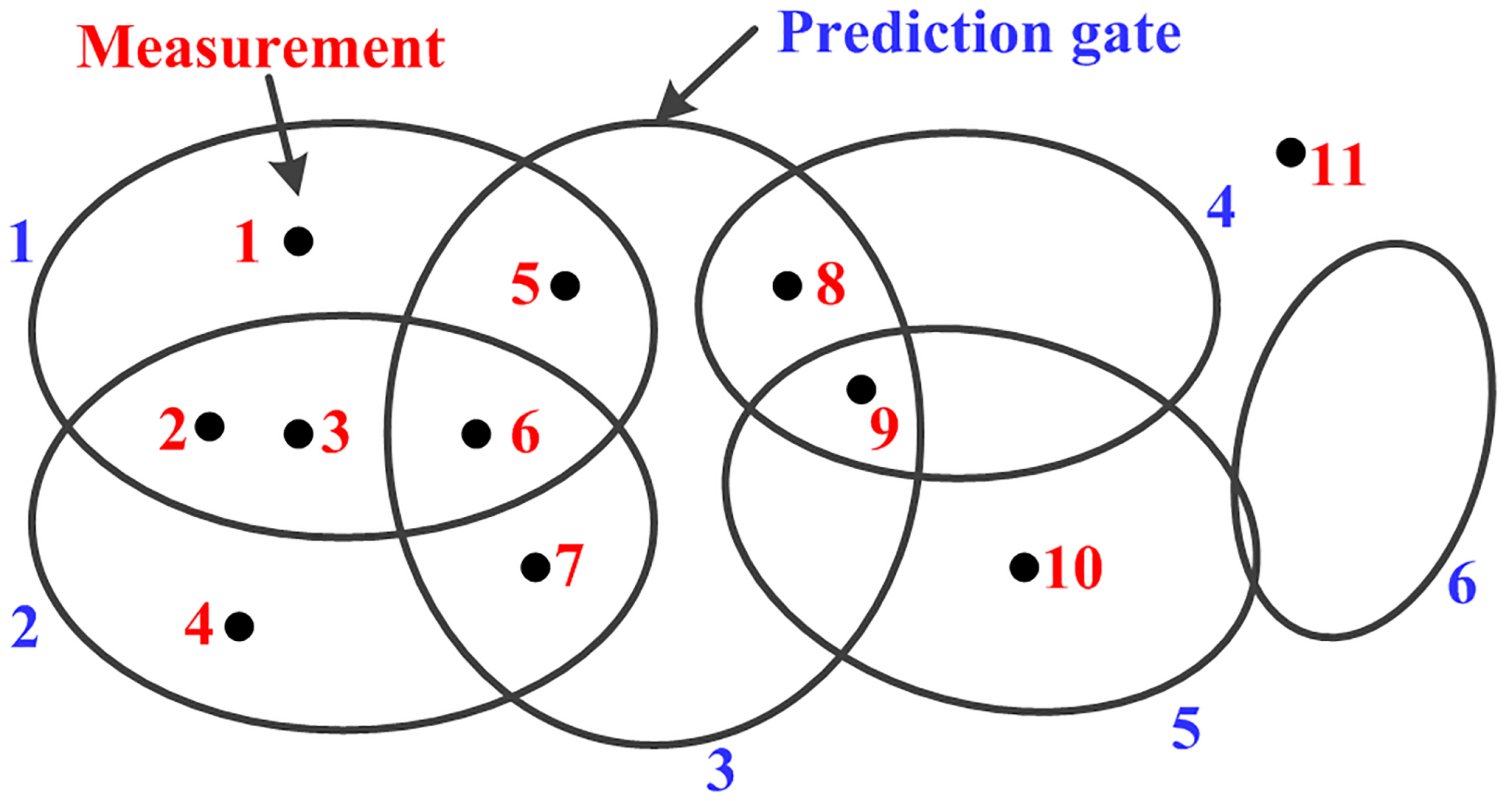
Interpretation of associations between measurements and prediction components.

All measurements that fulfill (59) are put into the same subset *W*. The measurements that do not fulfill (59) for any component, e.g., measurement 11 in Fig 3, are placed in individual subsets containing only one measurement. However, a measurement may fall into two or more prediction gates, e.g., measurements 2 and 3, which will generate multiple partitions. Thus prediction partition may still obtain a large number of partitions when the prediction gates corresponding to different sub-objects overlap seriously.

To further reduce the number of partitions, we consider selecting only the partitions with *η* largest probabilities from all possible partitions by using pseudo-likelihood method. Based on it, the calculation of all the pseudo-likelihoods reduces to calculate $N_{z,k}\times \sum_{i=1}^{N_{x,k}}n_{k}^{\left(i,s\right)}$ likelihoods (41g) with |*W*| = 1, which is simpler than to calculate $(2^{N_{z,k}}-1)\times \sum_{i=1}^{N_{x,k}}n_{k}^{\left(i,s\right)}$ true likelihoods (41g).

The interpretation of measurement set partitioning method in Table 2 is given as follows.

1) Line 2: *L*_*j*_ contains the prediction components whose gates the measurement $z_{k}^{\left(j\right)}$ falls into; *M*_*i*_ contains the measurements that fall into the gate of the component $\xi_{k|k-1}^{\left(i\right)}$; $\tilde{J}$ contains the measurements that fall into only one prediction gate, called unique-measurement, e.g., measurements 1 and 4 in Fig 3; $\bar{J}$ contains the measurements that fall into two or more prediction gates, called shared-measurement, e.g., measurements 2 and 5; $\tilde{I}$ contains the components that are associated with one or more unique-measurements, e.g., components 1, 2, and 5; $\bar{I}$ contains the components that are only associated with shared-measurements, e.g., components 3 and 4.

2) Because both the unique-measurements and the measurements not associated with any component have unique partitions, discussions are focused on the partitioning method of shared-measurements.

3) Line 4: if a shared-measurement (e.g., measurement 2) is associated with the components that are all contained by $\tilde{I}$, it can be independently put into the subset *W* corresponding to its any associated component.

4) Line 5–21: if two or more components included in $\bar{I}$(e.g., components 3 and 4) share one or more measurements, each partition for the measurements associated with these components (e.g., measurements 5–9) should guarantee that the subsets corresponding to these components are all non-empty.

5) Each *F*_*l*_ stores the measurements that should be partitioned interdependently, and *G*_*l*_ stores all feasible partitions of measurements in *F*_*l*_. Based on this, all possible partitions of the measurement set can be obtained easily.

Additionally, when the number of measurements is large, clustering technology is needed to simplify the association process by clustering *N*_***z***,*k*_ measurements into ${\bar{N}}_{z,k}({\bar{N}}_{z,k}\ll N_{z,k})$ clusters, each treated as a whole.

## Simulation results

This section presents numerical results of a simulation study that demonstrates the effectiveness of the proposed GGIW-PHD and MM-GGIW-PHD filters. Here, the improved optimal sub-pattern assignment (OSPA) distance given in [23] is used to evaluate the performance of tracking algorithms. Suppose that the true set of targets is

$$X_{k}={\left\{{\left\{\xi_{k}^{\left(j,s\right)}\right\}}_{s=1}^{n_{k}^{\left(j,s\right)}}\right\}}_{j=1}^{N_{x,k}},\xi_{k}^{\left(j,s\right)}≜(\gamma_{k}^{\left(j,s\right)},x_{k}^{\left(j,s\right)},X_{k}^{\left(j,s\right)})$$ (61)

The distance between a true sub-object $\xi_{k}^{\left(j,s\right)}$ and an estimated sub-object ${\hat{\xi }}_{k|k}^{\left(i,s\right)}$ is decomposed as

$$d(\xi_{k}^{\left(j,s\right)},{\hat{\xi }}_{k|k}^{\left(i,s\right)})=\frac{w_{\gamma }}{c_{\gamma }}d_{j,i,s}^{\left(c_{\gamma }\right)}+\frac{w_{x}}{c_{x}}d_{j,i,s}^{\left(c_{\text{x}}\right)}+\frac{w_{X}}{c_{X}}d_{j,i,s}^{\left(c_{X}\right)}$$ (62)

where *w*_*γ*_ + *w*_***x***_ *+ w*_***X***_ = 1, and

$$\left\{ \begin{array}{l} d_{j,i,s}^{\left(c_{\gamma }\right)}=\text{min}(c_{\gamma },\left|\gamma_{k}^{\left(j,s\right)}-{\hat{\gamma }}_{k|k}^{\left(i,s\right)}\right|)\\ d_{j,i,s}^{\left(c_{x}\right)}=\text{min}(c_{x},{\left\|x_{k}^{\left(j,s\right)}-{\hat{x}}_{k|k}^{\left(i,s\right)}\right\|}_{2})\\ d_{j,i,s}^{\left(c_{X}\right)}=\text{min}(c_{X},{\left\|X_{k}^{\left(j,s\right)}-{\hat{X}}_{k|k}^{\left(i,s\right)}\right\|}_{F}) \end{array}\right.$$ (63)

where |·| is absolute value, ||·||_2_ is Euclidean norm, and ||·||_*F*_ is Frobenius norm. The constants *c*_*γ*_, *c*_***x***_, and *c*_***X***_ are respectively chosen for the measurement rate, kinematic state, and extension state to satisfy the maximum expected error.

An optimal assignment $\bar{\pi }(j,s)$ of order *p* with cut-off *c* is given by

$$\begin{array}{l} \bar{\pi }(j,s)=\underset{\pi \in \prod_{{\hat{N}}_{x,k}}}{\text{arg}\;\text{min}}\sum_{j=1}^{N_{x,k}}\sum_{s=1}^{n_{k}^{\left(j,s\right)}}{\left(d_{j,i,s}^{\left(c\right)}\right)}^{p}\\ d_{j,i,s}^{\left(c\right)}=\text{min}(c,d(\xi_{k}^{\left(j,s\right)},{\hat{\xi }}_{k|k}^{\left(i,s\right)})) \end{array}$$ (64)

Then the tracking performance is presented in terms of the quantity as

$${\bar{d}}_{p}^{\left(c\right)}={\left(\frac{1}{{\hat{N}}_{x,k}}\left(\sum_{j=1}^{N_{x,k}}{\left(d_{j,\bar{\pi }(j,s)}^{\left(c\right)}\right)}^{p}+c^{p}({\hat{N}}_{x,k}-N_{x,k})\right)\right)}^{1/p}$$ (65)

### Modeling and target tracking setup

**Fig 4:**
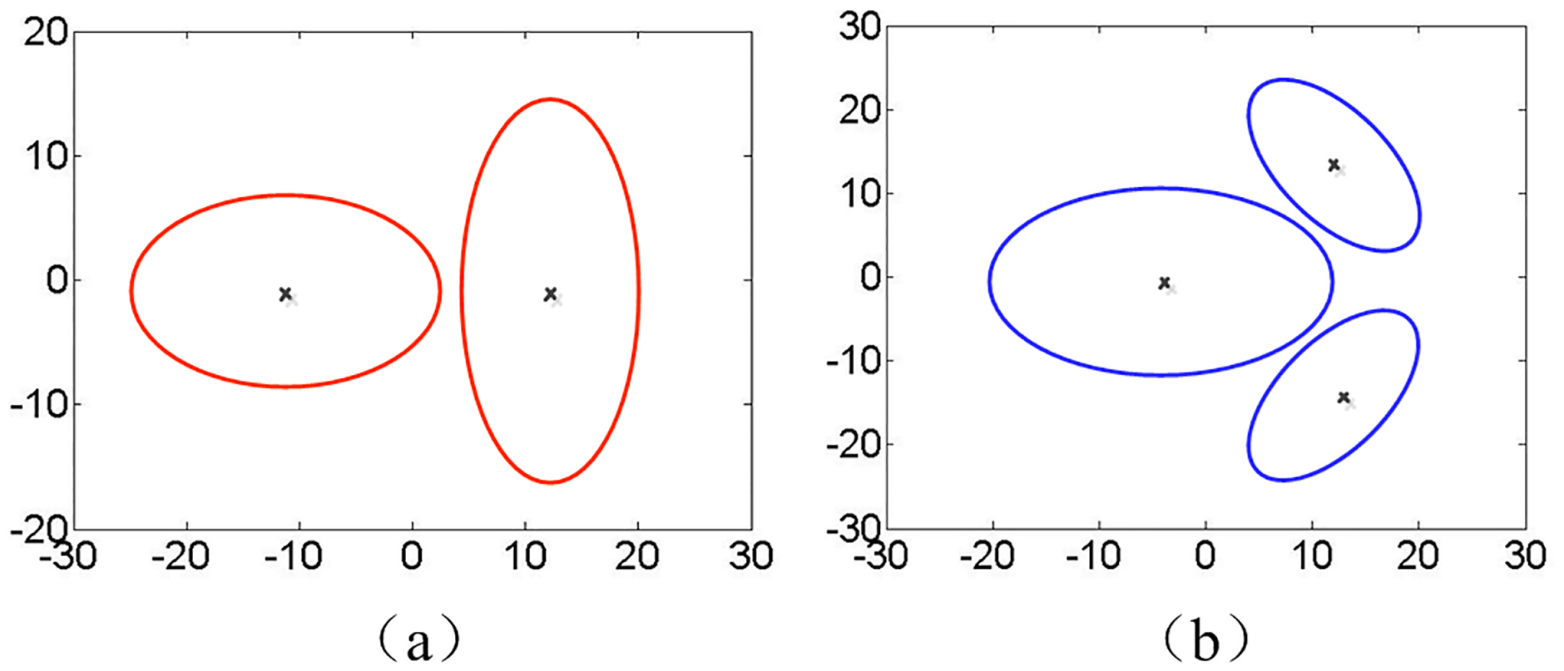
Simplified non-ellipsoidal extended target. T1; (b) T2.

Two types of non-ellipsoidal extended targets (T1 and T2) that simplified by several sub-ellipsoids are used in this simulation study, as shown in Fig 4.

We assume that the number of sub-objects for each extended target is known. The label scheme [22] is used to determine which sub-object a GGIW component belongs to. Besides, the profiles for each extended target are summarized in Table 3, such as size (A, a), orientation (***A***), and measurement rate (*γ*) of each sub-ellipsoid.

**Table 3:** Extension parameters (size, shape, orientation) and measurement rate for each ellipse.

No. of ellipse	Target T1					Target T2				
	A(m)	a(m)	*δ*	*A*	*γ*	A(m)	a(m)	*δ*	*A*	*γ*
One ellipse for GIW-PHD and GGIW-CPHD	28	18	30	$1/\sqrt{30}I_{d}$	30	26	24	26	$1/\sqrt{26}I_{d}$	40
Sub-ellipse 1	16	8	15	$1/\sqrt{15}I_{d}$	15	18	10	20	$1/\sqrt{20}I_{d}$	20
Sub-ellipse 2	14	8	15	$1/\sqrt{15}I_{d}$	15	$8\sqrt{2}$	$4\sqrt{2}$	10	$1/\sqrt{10}I_{d}$	10
Sub-ellipse 3						$8\sqrt{2}$	$4\sqrt{2}$	10	$1/\sqrt{10}I_{d}$	10

**Fig 5:**
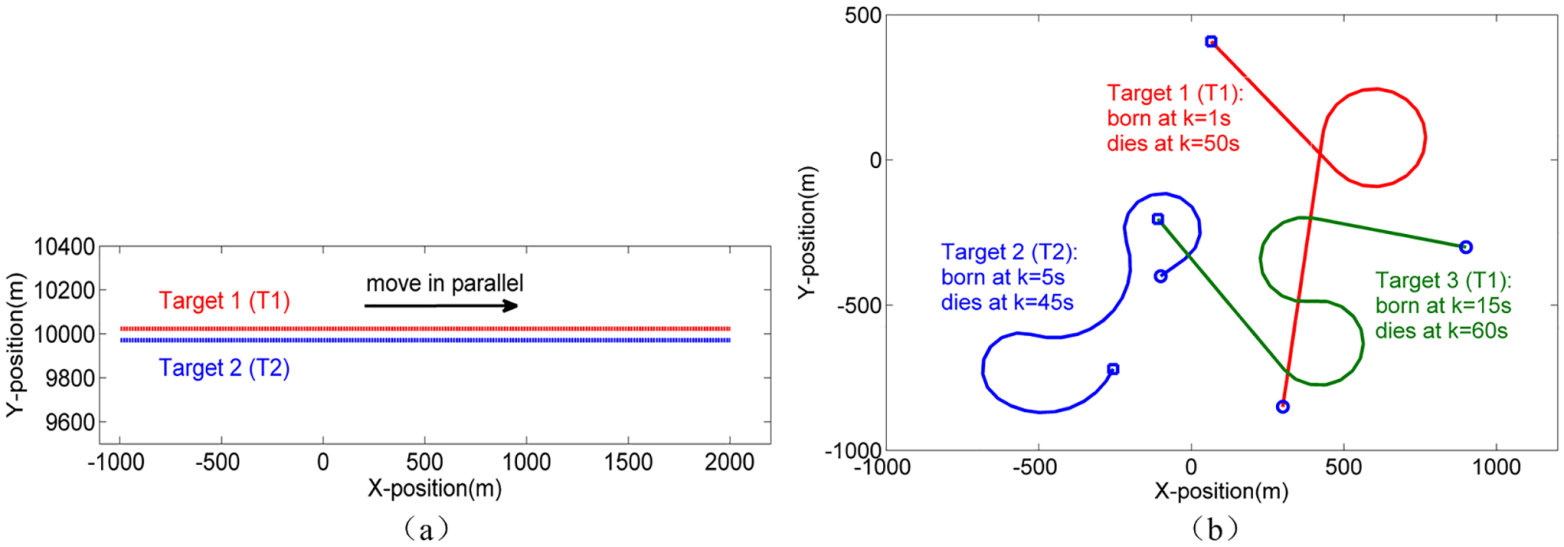
True target tracks used in simulations. (a) S1: Two non-maneuvering extended targets that move in parallel. (b) S2: Three maneuvering extended targets that appear and disappear at different times, the start/end positions for each target are denoted by **○/□**.

Two different scenarios are shown in Fig 5. The first scenario (S1): the tracking performances of the proposed GGIW-PHD filter, GIW-PHD filter and GGIW-CPHD filters are compared. The second scenario (S2): for MNETT, the performances of the proposed GGIW-PHD and MM-GGIW-PHD filters are compared. The true extension state of sub-object *s* of *i*th extended target is given by

$$X_{k}^{\left(i,s\right)}=A_{k}^{\left(i,s\right)}diag\left(\left[{\left({\text{A}}_{k}^{\left(i,s\right)}\right)}^{2},{\left(a_{k}^{\left(i,s\right)}\right)}^{2}\right]\right){\left(A_{k}^{\left(i,s\right)}\right)}^{\text{T}}$$ (66)

In S1, the two extended targets (T1 and T2) start from $x_{0}^{1}={\left[-1000m,10023m,100m/s,0,0,0\right]}^{T}$ and $x_{0}^{2}={\left[-1000m,9971m,100m/s,0,0,0\right]}^{T}$ with a constant speed of 100m/s, the sampling period *T* = 1s, *θ* = 1s, and no process noise added. The measurements of each sub-object are generated in terms of the measurement model (10) with Gaussian measurement noise *N*(0, ***R***_*k*_) with ***R***_*k*_ = *diag*([9,9])m^2^. The number of measurements for each sub-object yields to Poisson distribution with rate *γ* given in Table 3. There are 10 clutter measurements per time step.

$$A^{r}=\left[\begin{array}{cc} \text{cos}\theta^{r} & -\text{sin}\theta^{r}\\ \text{sin}\theta^{r} & \text{cos}\theta^{r} \end{array}\right]\text{with}\left[\begin{array}{c} \theta^{2}=\pi /15\;\text{rad}\\ \theta^{3}=-\pi /15\;\text{rad} \end{array}\right].$$

In S2, the three maneuvering extended targets respectively start from

$$\begin{array}{c} x_{0}^{1}={\left[300\text{m},-850\text{m},7\text{m}/\text{s},50\text{m}/\text{s},0,0\right]}^{\text{T}}\\ x_{0}^{2}={\left[-100\text{m},-400\text{m},40\text{m}/\text{s},30\text{m}/\text{s},0,0\right]}^{\text{T}}\\ x_{0}^{3}={\left[900\text{m},-300\text{m},-50\text{m}/\text{s},10\text{m}/\text{s},0,0\right]}^{\text{T}} \end{array}$$

The number of clutter measurements is the same as that in S1. The true measurement noise yields to *N*(0, ***R***_*k*_) with ***R***_*k*_ = *diag*([1,1])m^2^. The sampling period *T* = 1s, and *θ* = 1s. All sub-object models *m*^(*i*,*s*)^(*s* = 1,…,*n*^(*i*,*s*)^, *i* = 1,2,3) are given in Table 4. When tracking maneuvering extended targets, the proposed GGIW-PHD filter with a low process noise may obtain unacceptable estimates, which, but, can be prevented by increasing the process noise properly. Therefore, a lower (*σ* = 0.1) and a higher (*σ* = 1) process noises are considered in this simulation. For the proposed MM-GGIW-PHD filter, the overall models ${\bar{m}}^{r}$(r=1,2,3) are given in Table 5, where ${\bar{m}}^{2}$ and ${\bar{m}}^{3}$ are maneuver models. According to (50), the combined models for all sub-objects are given in Table 6. Additionally, the model transition probabilities *t*(*r*|*r*′)(*r*,*r*′ = 1,2,3) in (51) are designed as *t*(*r*|*r*) = 0.8 and *t*(*r*|*r*′) = 0.1(*r* ≠ *r*′), ∀*r*,*r*′ = 1,2,3. The birth intensities $\pi_{}^{r}$ (r=1,2,3) in (52) are designed as *π* = [0.8 0.1 0.1].

**Table 4:** Individual models.

	*m*^(1,1)^,*m*^(1,2)^,*m*^(3,1)^,*m*^(3,2)^	*m*^(2,1)^	*m*^(2,2)^,*m*^(2,3)^
∑^(i,s)^	*σ*	*σ*	*σ*
*δ*^(i,s)^	15	20	10
***A***^(i,s)^	$1/\sqrt{15}I_{d}$	$1/\sqrt{20}I_{d}$	$1/\sqrt{10}I_{d}$

*σ* = 0.1 and 1 for a lower and higher process noise, respectively.

**Table 5:** Overall models.

	${\bar{m}}^{1}$	${\bar{m}}^{2}$	${\bar{m}}^{3}$
${\bar{\Sigma }}^{r}$	1	1/*σ*	1/*σ*
${\bar{\delta }}^{r}$	3	3	3
${\bar{A}}^{r}$	$1/\sqrt{3}I_{d}$	$1/\sqrt{3}A^{2}$	$1/\sqrt{3}A^{3}$

**Table 6:** Combined models for MM-GGIW-PHD filter.

	*m*^(1,1)^,*m*^(1,2)^,*m*^(3,1)^,*m*^(3,2)^			*m*^(2,1)^			*m*^(2,2)^,*m*^(2,3)^		
	∑^(*i*,*s*|*r*)^	*δ*^(*i*,*s*|*r*)^	***A***^(*i*,*s*|*r*)^	∑^(2,1|*r*)^	*δ*^(2,1|*r*)^	***A***^(2,1|*r*)^	∑^(*i*,*s*|*r*)^	*δ*^(*i*,*s*|*r*)^	***A***^(*i*,*s*|*r*)^
*m*^(*i*,*s*|1)^	*σ*	15	***$1/\sqrt{15}I_{d}$***	*σ*	20	***$1/\sqrt{20}I_{d}$***	*σ*	10	***$1/\sqrt{10}I_{d}$***
*m*^(*i*,*s*|2)^	1	15	$1/\sqrt{15}A^{2}$	1	20	$1/\sqrt{20}A^{2}$	1	10	$1/\sqrt{10}A^{2}$
*m*^(*i*,*s*|3)^	1	15	$1/\sqrt{15}A^{3}$	1	20	$1/\sqrt{20}A^{3}$	1	10	$1/\sqrt{10}A^{3}$

The survival probability is *p*_*S*_ = 0.95, and detection probability is *p*_*D*_ = 0.98. The parameters for OSPA distance are set to *p* = 2, *c*_*γ*_ = 20, *c*_***x***_ = 60, *c*_***X***_ = 100, *c* = *c*_*γ*_ + *c*_***x***_ + *c*_***X***_, *w*_*γ*_ = 0.1, *w*_***x***_ = 0.8, and *w*_***X***_ = 0.1. The number of Monte Carlo simulations is 100. The parameters of birth components are set as follows

$$w_{b,k}^{\left(i,s\right)}=0.1,m_{b,k}^{\left(i,s\right)}={\left[{\left(x_{0}^{\left(i,s\right)}\right)}^{\text{T}}\;0_{4}^{\text{T}}\right]}^{\text{T}}$$

$$P_{b,k}^{\left(i,s\right)}=diag([10^{2}m^{2}\;10^{2}m^{2}/s^{2}\;0.1^{2}m^{2}/s^{4}])$$

$$\alpha_{b,k}^{\left(i,s\right)}=0.04,\beta_{b,k}^{\left(i,s\right)}=0.01$$

$$v_{b,k}^{\left(i,s\right)}=7,V_{b,k}^{\left(i,s\right)}=diag([20^{2}\;20^{2}])$$

The mean vectors $m_{b,k}^{\left(i,s\right)}$ are set to the true starting points of sub-objects. Additionally, pruning and merging are performed at each time step with *T* = 10^−3^, *U* = 0.6, and *J*_max_ = 1000.

### Two extended targets that move in parallel: S1

**Fig 6:**
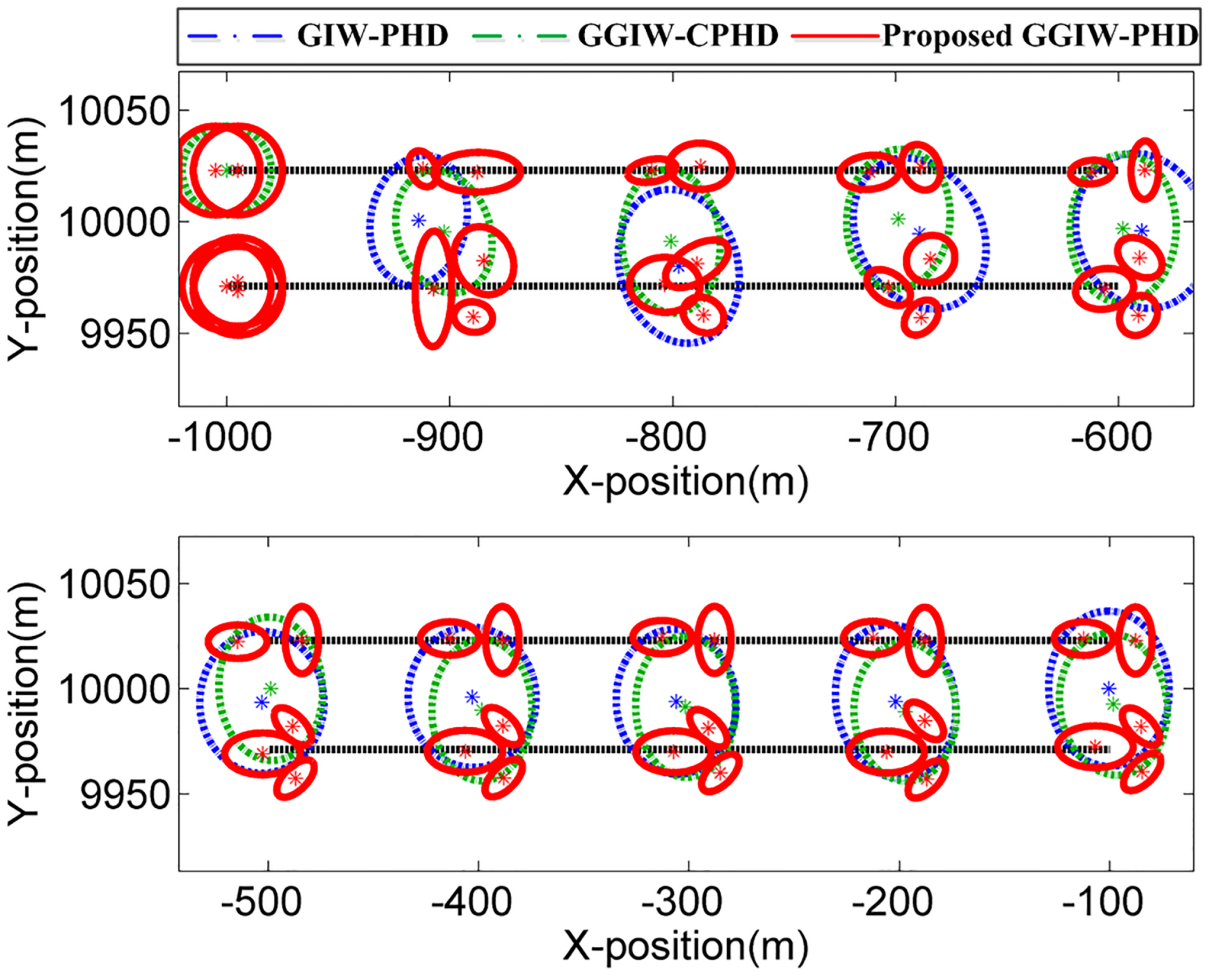
Extended target tracking results in S1.

**Fig 7:**
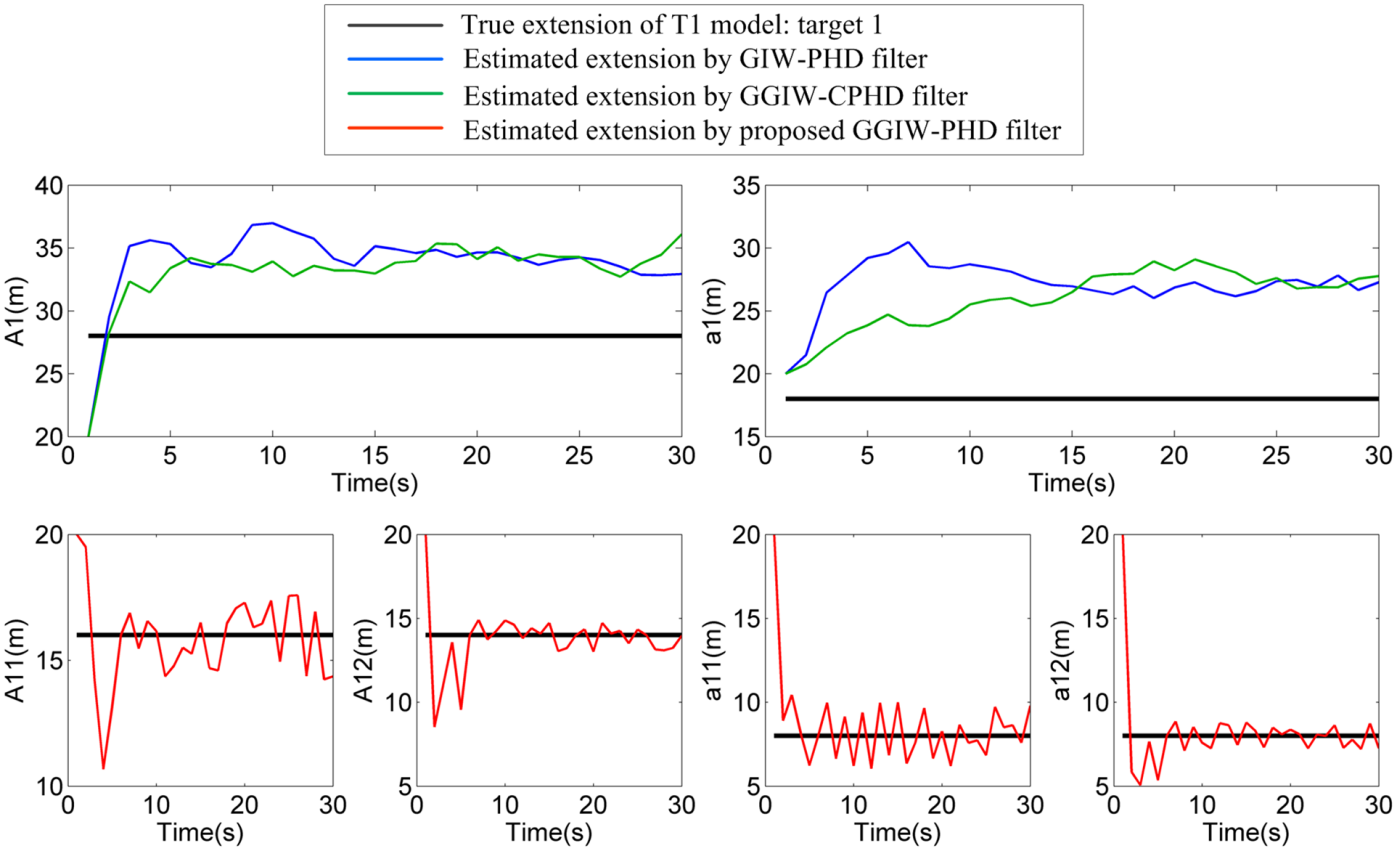
Estimates of the major and minor axes of target 1.

**Fig 8:**
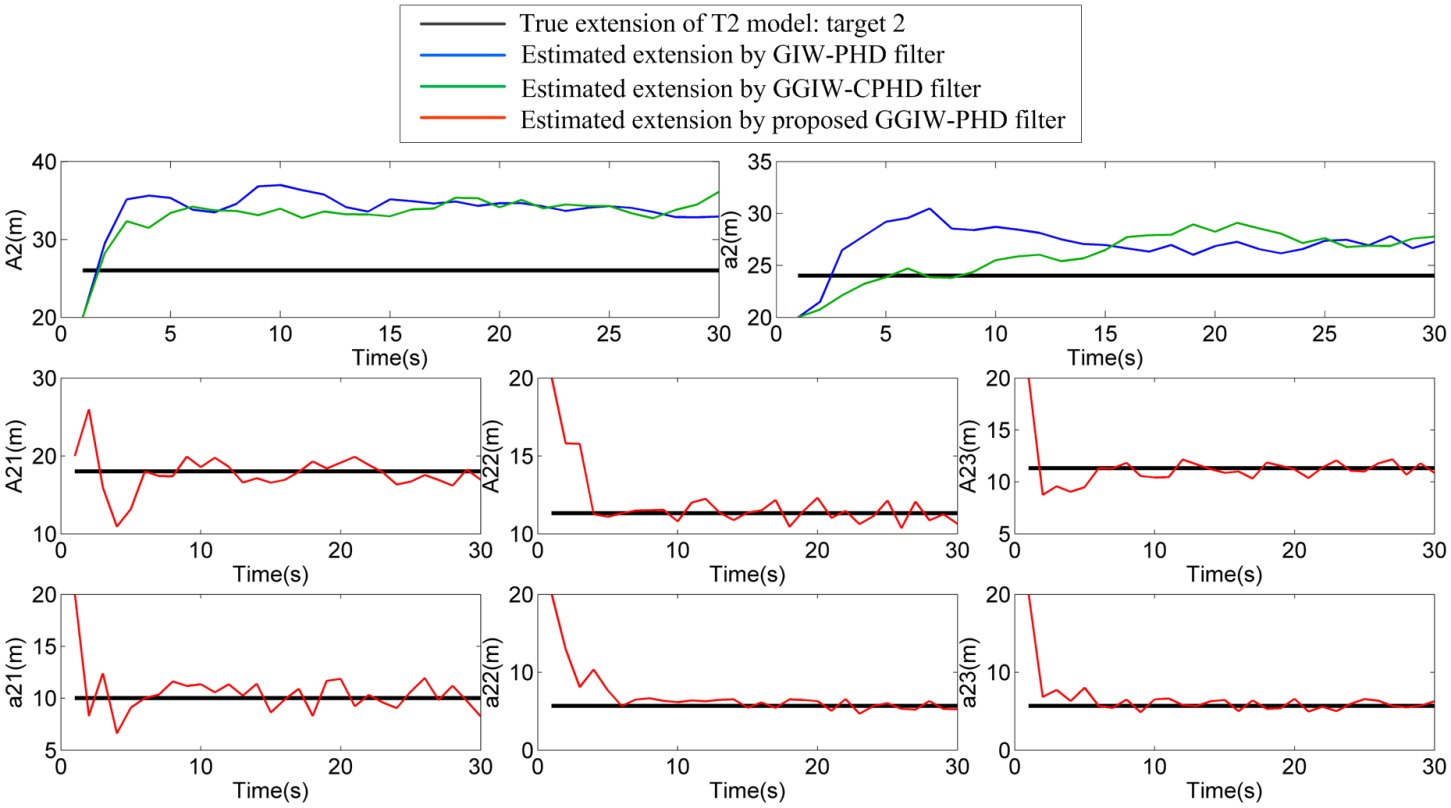
Estimates of the major and minor axes of target 2.

In this scenario, the two extended targets (T1 and T2) move in parallel, and the true target extensions’ standard deviation ellipsoids are separated by 10m. For space consideration, the filter output for only first ten sampling periods in a single run is shown in Fig 6 where the estimated positions and extensions of extended targets by GIW-PHD and GGIW-CPHD filters and of sub-objects by proposed GGIW-PHD filter are given. Besides, estimates of the major and minor axes for two extended targets are shown in Figs 7 and 8. As shown in Fig 6, the proposed GGIW-PHD filter can obtain detailed extension information about size, shape, and orientation, while GIW-PHD and GGIW-CPHD filters only can approximate the extension by using an ellipsoid (almost a circle) without shape and orientation information. In addition, GIW-PHD and GGIW-CPHD filters incorrectly obtain one large extended target, instead of two smaller ones, in the most majority of sampling periods. This is what causes that the estimates of the major and minor axes by these two filters are much longer than the true ones, as shown in Figs 7 and 8. The proposed GGIW-PHD filter can almost accurately estimate the major and minor axes for each sub-object. The reason is the use of prediction partition with pseudo-likelihood method rather than distance partition.

**Fig 9:**
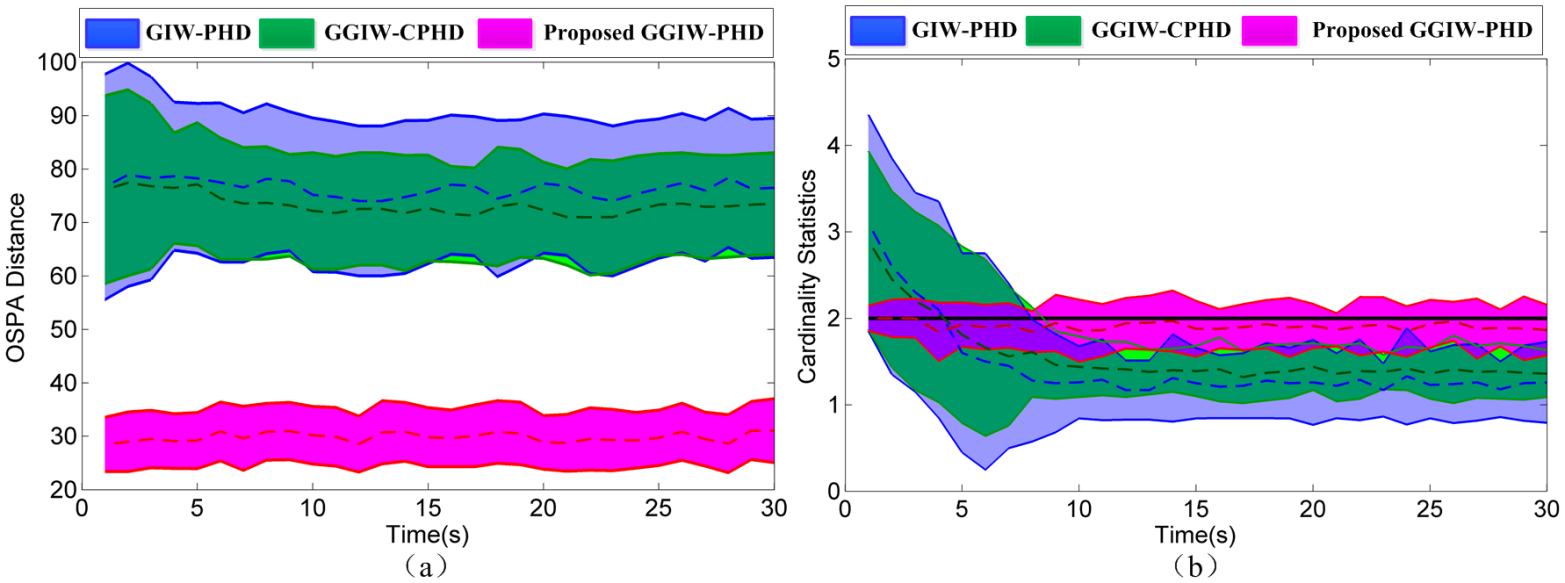
The comparison of tracking performance in S1. (a) OSPA distances: mean (dashed lines)±one standard deviation. (b) Cardinality estimates: mean (dashed lines)±one standard deviation.

The OSPA distances and cardinality estimates are shown in Fig 9. Because of estimating one large target instead of two true smaller ones, GIW-PHD and GGIW-CPHD filters have deteriorating estimation performances. Thus, compared to these two filters, the proposed GGIW-PHD filter has smaller OSPA and cardinality errors on average, as shown in Fig 9. GGIW-CPHD filter has smaller cardinality errors than GIW-PHD filter, because the former propagates the entire cardinality distribution, whereas the latter propagates only the cardinality mean.

### Three maneuvering extended targets: S2

**Fig 10:**
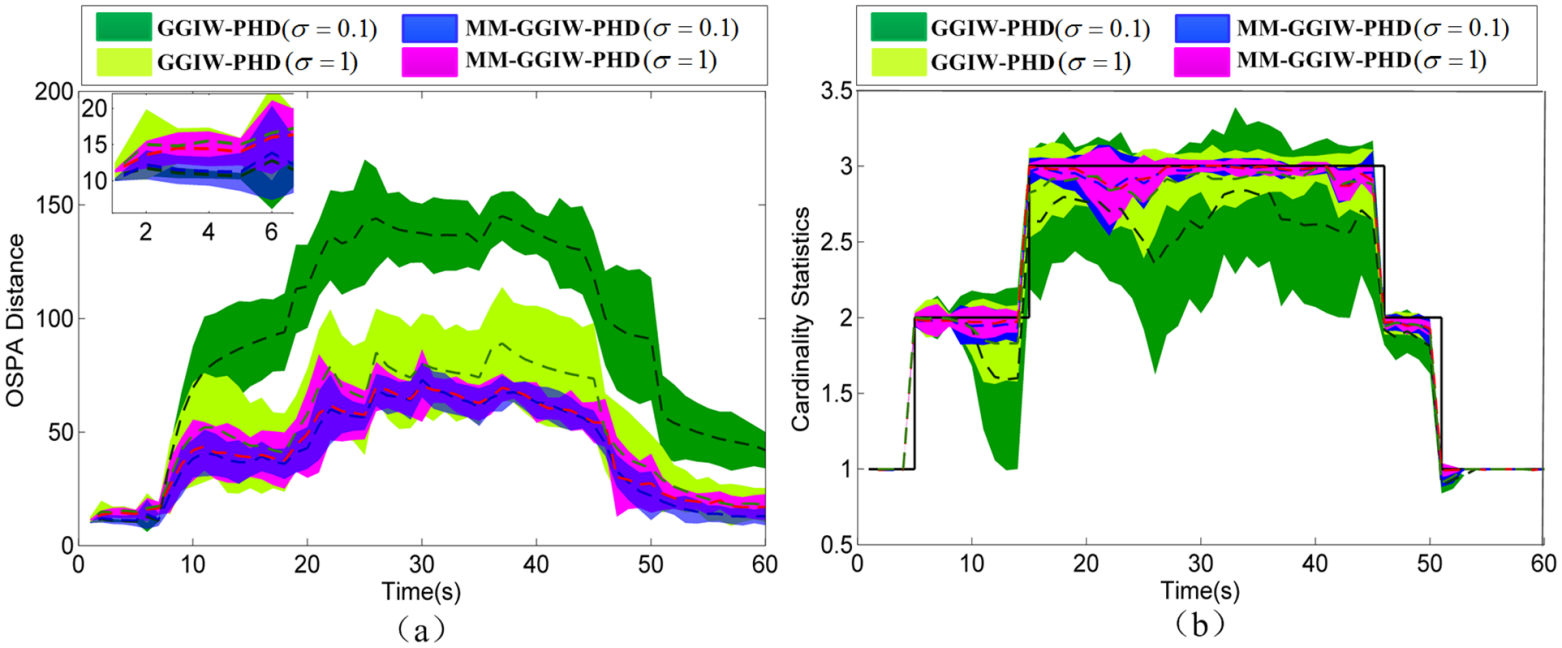
The comparison of tracking performance in S2. (a) OSPA distances: mean (dashed lines)±one standard deviation. (b) Cardinality estimates: mean (dashed lines)±one standard deviation.

In this scenario, three extended targets (modeled by T1, T2, and T1 respectively) implement maneuver flight. Fig 10 shows OSPA distances and cardinality estimates of the proposed GGIW-PHD and MM-GGIW-PHD filters, all with a lower (*σ* = 0.1) and a higher (*σ* = 1) process noises.

As shown in Fig 10, when the maneuver starts (Target 2 at *k* = 8s), the OSPA distances and cardinality errors of GGIW-PHD filter with a lower process noise of *σ* = 0.1 increase drastically. Also, during and after the maneuver for all extended targets, GGIW-PHD filter has larger OSPA distances and cardinality errors, compared to MM-GGIW-PHD filter with the same noise. By increasing the process noise, the tracking performance of GGIW-PHD filter is improved greatly. Nevertheless, with a higher process noise of *σ* = 1, the MM-GGIW-PHD filter still has better tracking performance than that of GGIW-PHD filter. During maneuver, two MM-GGIW-PHD filters with different process noises have similar performance. However, in non-maneuvering conditions (*k* = 1s ~ 7s and 46s ~ 50s), the OSPA distances of MM-GGIW-PHD filter with *σ* = 0.1 are smaller than that of MM-GGIW-PHD filter with *σ* = 1. Thus, the proposed MM-GGIW-PHD filter has good performance for both non-maneuvering and maneuvering conditions.

**Fig 11:**
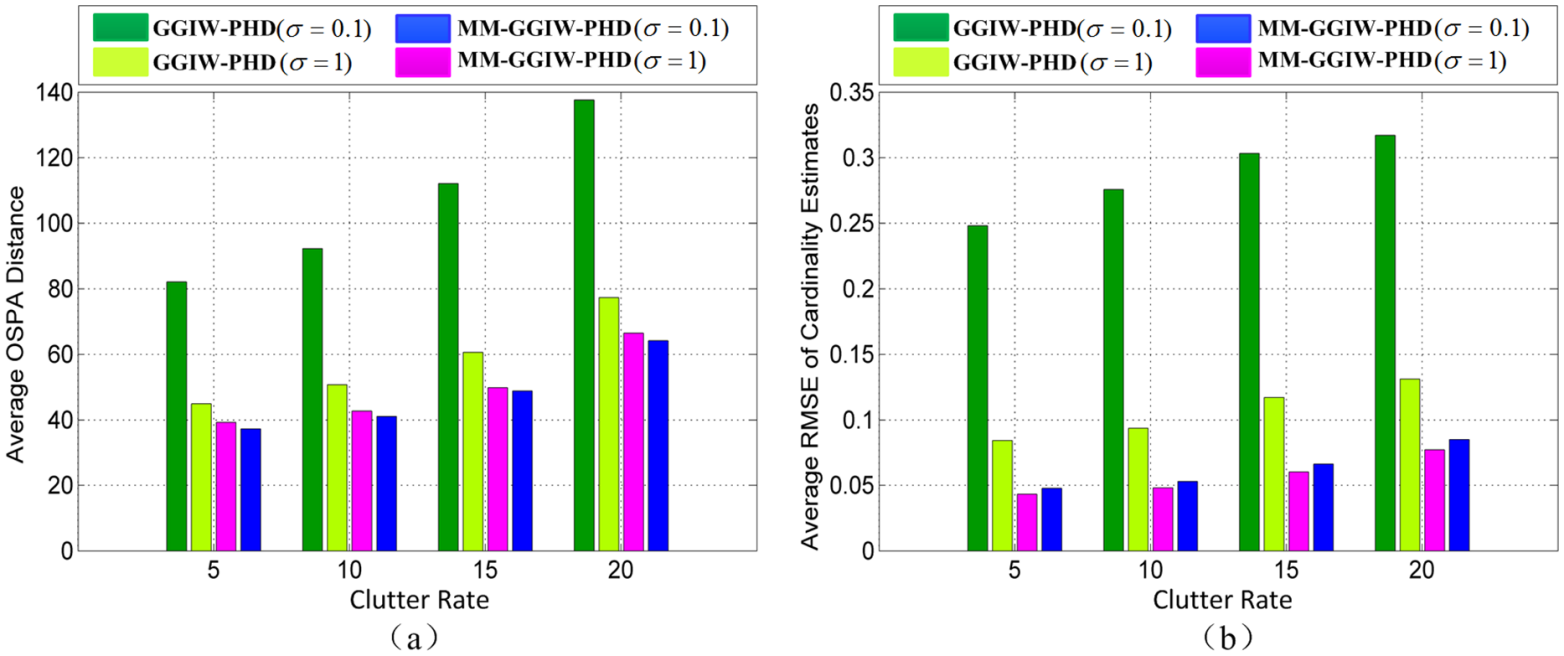
(a) Average OSPA distances against the clutter rate. (b) Average RMSEs of cardinality estimates against the clutter rate.

Fig 11(a) is the result of average OSPA distances at different clutter rates. Fig 11(b) shows average root-mean-square errors (RMSEs) of cardinality estimates. We can see that the tracking accuracies of all filters reduce, along with the increasing of clutter rate. At different clutter rates, MM-GGIW-PHD filter with *σ* = 0.1 always has best tracking performance among all filters with different process noises. This also demonstrates the effectiveness of MM-GGIW-PHD filter.

## Conclusions and future work

For NETT and MNETT problems, this paper approximates the target extension by multiple ellipsoids, each represented by a random matrix. Based on this, an improved GGIW-PHD filter and a MM-GGIW-PHD filter are proposed. Actually, when treated as a whole, a target group can be regarded as an extended target. Thus, the proposed approaches can be applied to GTT with little modification.

To obtain a bounded and integrative component-merging criterion, a new one ranging from 0 to 1 is derived based on Hellinger distance. Additionally, a new merging method is proposed by using moment matching method, instead of using the weighted mean of components as the merging result.

To partition the measurement set, a specific implementation of prediction partition with pseudo-likelihood method is presented. It is important to obtain all feasible partitions and reduce computational complexity.

Actually, the number of sub-objects of an extended target should be time-varying to approximate the true extension, especially during maneuver. Thus, how to adaptively determine the number of sub-objects is a topic for our future work. In addition, the proposed models and approaches will be incorporated into more advanced algorithms in our future research.

## Supporting information

S1 File Derivation of the updating PHD. (PDF)

S2 File Derivation of the component-merging method. (PDF)
