# Supplementary material for: Improved GGIW-PHD filter for maneuvering non-ellipsoidal extended targets or group targets tracking based on sub-random matrices
Source: PLoS One. 2018 Feb 14;13(2):e0192473. doi: 10.1371/journal.pone.0192473 (PMC5812665; doi:10.1371/journal.pone.0192473)
Supplement: S1 File — (PDF) [file pone.0192473.s001.pdf]

## Derivation of the updating PHD

Under the measurement model (10), the likelihood of $n$ measurements $z_i$ is

$$\prod_{i=1}^{n}\phi_z(\xi)=\prod_{i=1}^{n}\mathcal{N}(z_i;(H\otimes I_d)x,BXB^{\mathrm{T}})$$

$$=(2\pi)^{-nd/2}\left|BXB^{\mathrm{T}}\right|^{-n/2}etr\left(-\frac{1}{2}\left(\sum_{i=1}^{n}(z_i-\tilde{H}x)(z_i-\tilde{H}x)^{\mathrm{T}}\right)(BXB^{\mathrm{T}})^{-1}\right) \quad \text{(A1)}$$

where $\tilde{H}=H\otimes I_d$. Define

$$\overline{z}\triangleq(1/n)\sum_{i=1}^{n}z_i,\quad Z\triangleq\sum_{i=1}^{n}(z_i-\overline{z})(z_i-\overline{z})^{\mathrm{T}} \quad \text{(A2)}$$

then the summation can be rewritten as

$$\sum_{i=1}^{n}(z_i-\tilde{H}x)(z_i-\tilde{H}x)^{\mathrm{T}}=Z+n(\overline{z}-\tilde{H}x)(\overline{z}-\tilde{H}x)^{\mathrm{T}} \quad \text{(A3)}$$

Based on this, Equation (A1) can be converted to

$$\prod_{i=1}^{n}\phi_z(\xi)=(2\pi)^{-nd/2}\left|BXB^{\mathrm{T}}\right|^{-n/2}etr\left(-\frac{1}{2}Z(BXB^{\mathrm{T}})^{-1}\right)etr\left(-\frac{1}{2}(\overline{z}-\tilde{H}x)(\overline{z}-\tilde{H}x)^{\mathrm{T}}\left(BXB^{\mathrm{T}}/n\right)^{-1}\right) \quad \text{(A4a)}$$

$$=(2\pi)^{-(n-1)d/2}\left|BXB^{\mathrm{T}}\right|^{-(n-1)/2}n^{-d/2}etr\left(-\frac{1}{2}Z(BXB^{\mathrm{T}})^{-1}\right)\mathcal{N}\left(\overline{z};\tilde{H}x,BXB^{\mathrm{T}}/n\right) \quad \text{(A4b)}$$

$$=\mathcal{L}_{aux}\mathcal{N}\left(\overline{z};\tilde{H}x,BXB^{\mathrm{T}}/n\right) \quad \text{(A4c)}$$

Let the prediction PHD component be

$$\mathcal{GGIW}(\xi;\zeta)=\mathcal{G}(\gamma;\alpha,\beta)\mathcal{N}(x;m,P\otimes X)\mathcal{IW}(X;v,V) \quad \text{(A5)}$$

thus

$$e^{-\gamma}\cdot\gamma^{|W|}\cdot\prod_{i=1}^{n}\phi_z(\xi)\cdot\mathcal{GGIW}(\xi;\zeta)$$

$$=\mathcal{L}_{aux}\mathcal{IW}(X;v,V)e^{-\gamma}\cdot\gamma^{|W|}\cdot\mathcal{G}(\gamma;\alpha,\beta)\mathcal{N}\left(\overline{z};\tilde{H}x,BXB^{\mathrm{T}}/n\right)\mathcal{N}(x;m,P\otimes X) \quad \text{(A6a)}$$

$$=\mathcal{L}_{aux}\mathcal{IW}(X;v,V)\frac{\beta^{\alpha}}{\beta_{+}^{\alpha_{+}}}\frac{\Gamma(\alpha_{+})}{\Gamma(\alpha)}\mathcal{G}(\gamma;\alpha_{+},\beta_{+})\mathcal{N}(x;m_{+},P_{+}\otimes X)\mathcal{N}\left(\overline{z};\tilde{H}m,SX\right) \quad \text{(A6b)}$$

where

$$\alpha_{+}=\alpha+\left|W\right|,\quad \beta_{+}=\beta+1 \quad \text{(A6c)}$$

$$m_{+}=m+(K\otimes I_{d})(\bar{z}-\tilde{H}m) \tag{A6d}$$

$$P_{+}=P-KSK^{\mathrm{T}} \tag{A6e}$$

with

$$S=HPH^{\mathrm{T}}+(1/n)\left|B\right|^{2/d} \tag{A6f}$$

$$K=PH^{\mathrm{T}}S^{-1} \tag{A6g}$$

Here, there is an assumption that $BXB^{\mathrm{T}}\approx\left|B\right|^{2/d}X$, based on which the result (A6) can be obtained easily by using some calculation rules of Kronecker product.

Except the obtained updating gamma and Gaussian distributions, the rest of (A6b) can be derived as follows

$$\frac{\beta^{\alpha}}{\beta_{+}{}^{\alpha_{+}}}\frac{\Gamma(\alpha_{+})}{\Gamma(\alpha)}\mathcal{L}_{aux}\mathcal{N}\left(\bar{z};\tilde{H}m,SX\right)\mathcal{IW}(X;v,V)$$

$$=\frac{\beta^{\alpha}}{\beta_{+}{}^{\alpha_{+}}}\frac{\Gamma(\alpha_{+})}{\Gamma(\alpha)}(2\pi)^{-(n-1)d/2}\left|BXB^{\mathrm{T}}\right|^{-(n-1)/2}n^{-d/2}(2\pi)^{-d/2}\left|SX\right|^{-1/2}\frac{2^{-(v-d-1)d/2}\left|V\right|^{(v-d-1)/2}}{\Gamma_{d}\left((v-d-1)/2\right)\left|X\right|^{v/2}}$$

$$\times etr\left(-\frac{1}{2}(\bar{z}-\tilde{H}m)(\bar{z}-\tilde{H}m)^{\mathrm{T}}(SX)^{-1}\right)etr\left(-\frac{1}{2}X^{-1}V\right)etr\left(-\frac{1}{2}Z(BXB^{\mathrm{T}})^{-1}\right) \tag{A7a}$$

$$=\frac{\beta^{\alpha}}{\beta_{+}{}^{\alpha_{+}}}\frac{\Gamma(\alpha_{+})}{\Gamma(\alpha)}(2\pi)^{-nd/2}\left|B\right|^{-(n-1)}(nS)^{-d/2}$$

$$\times\frac{2^{-(v-d-1)d/2}\Gamma_{d}\left((v+n-d-1)/2\right)}{\Gamma_{d}\left((v-d-1)/2\right)}\cdot\frac{2^{(v+n-d-1)d/2}\left|V\right|^{(v-d-1)/2}}{\left|V+N+B^{-1}ZB^{-\mathrm{T}}\right|^{(v+n-d-1)/2}}$$

$$\times\frac{2^{-(v+n-d-1)d/2}\left|V+N+B^{-1}ZB^{-\mathrm{T}}\right|^{(v+n-d-1)/2}}{\Gamma_{d}\left((v+n-d-1)/2\right)\left|X\right|^{(v+n)/2}}\cdot etr\left(-\frac{1}{2}(V+N+B^{-1}ZB^{-\mathrm{T}})X^{-1}\right) \tag{A7b}$$

$$=\frac{\beta^{\alpha}}{\beta_{+}{}^{\alpha_{+}}}\frac{\Gamma(\alpha_{+})}{\Gamma(\alpha)}(\pi^{n}nS)^{-d/2}\left|B\right|^{-(n-1)}\cdot\frac{\left|V\right|^{(v-d-1)/2}}{\left|V_{+}\right|^{(v_{+}-d-1)/2}}\frac{\Gamma_{d}\left((v_{+}-d-1)/2\right)}{\Gamma_{d}\left((v-d-1)/2\right)}\times\mathcal{IW}(X;v_{+},V_{+}) \tag{A7c}$$

$$=\mathcal{L}\times\mathcal{IW}(X;v_{+},V_{+}) \tag{A7d}$$

where

$$v_{+} = v + n \tag{A7e}$$

$$V_{+} = V + N + B^{-1} Z B^{-\mathrm{T}} \tag{A7f}$$

$$N = S^{-1} (\bar{z} - \tilde{H} m)(\bar{z} - \tilde{H} m)^{\mathrm{T}} \tag{A7g}$$

The likelihood function $\mathcal{L}$ is given by

$$\mathcal{L} = (\pi^{n} n S)^{-d/2} \left|B\right|^{-(n-1)} \frac{\beta^{\alpha}}{\beta_{+}{}^{\alpha_{+}}} \frac{\Gamma(\alpha_{+})}{\Gamma(\alpha)} \frac{\left|V\right|^{(v-d-1)/2}}{\left|V_{+}\right|^{(v_{+}-d-1)/2}} \frac{\Gamma_{d}\left(\left(v_{+} - d - 1\right)/2\right)}{\Gamma_{d}\left(\left(v - d - 1\right)/2\right)} \tag{A7h}$$
