# Supplementary material for: Improved GGIW-PHD filter for maneuvering non-ellipsoidal extended targets or group targets tracking based on sub-random matrices
Source: PLoS One. 2018 Feb 14;13(2):e0192473. doi: 10.1371/journal.pone.0192473 (PMC5812665; doi:10.1371/journal.pone.0192473)
Supplement: S2 File — (PDF) [file pone.0192473.s002.pdf]

Derivation of the component-merging method

Here, the moment matching method is used to provide a convenient solution to the following GGIW component-merging problem.

$$\sum_{i=1}^{N} w_i \mathcal{G}(\gamma;\alpha_i,\beta_i)\mathcal{N}(x;m_i,P_i\otimes X)\mathcal{IW}(X;v_i,V_i) \quad \text{(B1a)}$$

$$\approx \bar{w}\mathcal{G}(\gamma;\alpha,\beta)\mathcal{N}(x;m,P\otimes X)\mathcal{IW}(X;v,V) \quad \text{(B1b)}$$

where the parameters $\alpha,\beta,m,P,v,$ and V need to be determined.

Because γ is a scalar and $x\in\mathbb{R}^{(sd)}$ is a vector while $X\in\mathbb{S}_{++}^{d}$ is a matrix, moment matching is more difficult than in a typical matching solution. So, use the following stacked matrix for moment matching

$$X^e \triangleq \begin{bmatrix} \gamma^e \\ x^e \\ X \end{bmatrix}, \begin{cases} \gamma^e \triangleq \left[\gamma, 0_{1\times(d-1)}\right] \\ x^e \triangleq \left[x, 0_{(sd)\times(d-1)}\right] \end{cases} \quad \text{(B2)}$$

1) Moments of state variables with an unweighted distribution: that is, considering a GGIW distribution

$$\mathcal{G}(\gamma;\alpha,\beta)\mathcal{N}(x;m,P\otimes X)\mathcal{IW}(X;v,V) \quad \text{(B3)}$$

the first two moments of X^e defined by (B3) are

$$\bar{X}^e \triangleq E\{X^e\} = \begin{bmatrix} E\{\gamma^e\} \\ E\{x^e\} \\ E\{X\} \end{bmatrix} = \begin{bmatrix} \left[E\{\gamma\}, 0_{1\times(d-1)}\right] \\ \left[E\{x\}, 0_{(sd)\times(d-1)}\right] \\ E\{X\} \end{bmatrix} = \begin{bmatrix} \bar{\gamma}^e \\ \bar{x}^e \\ \bar{X} \end{bmatrix} = \begin{bmatrix} \left[\bar{\gamma}=\alpha/\beta, 0_{1\times(d-1)}\right] \\ \left[\bar{x}=m, 0_{(sd)\times(d-1)}\right] \\ V/(v-2d-2) \end{bmatrix} \quad \text{(B4)}$$

$$P^e \triangleq E\{(X^e-\bar{X}^e)(\cdot)^{\mathrm{T}}\} = \begin{bmatrix} P_{11}^e & P_{12}^e & P_{13}^e \\ (P_{12}^e)^{\mathrm{T}} & P_{22}^e & P_{23}^e \\ (P_{13}^e)^{\mathrm{T}} & (P_{23}^e)^{\mathrm{T}} & P_{33}^e \end{bmatrix} \quad \text{(B5a)}$$

where $(\cdot)$ stands for the term right before it, and

$$P_{11}^e \triangleq P^{\gamma} = E\{(\gamma^e-\bar{\gamma}^e)(\cdot)^{\mathrm{T}}\} = E\{(\gamma-\alpha/\beta)(\cdot)^{\mathrm{T}}\} = \int(\gamma-\alpha/\beta)(\cdot)^{\mathrm{T}}\mathcal{G}(\gamma;\alpha,\beta)\,\mathrm{d}\gamma = \alpha/\beta^2 \quad \text{(B5b)}$$

$$P_{12}^{e} \triangleq E\{(\gamma^{e}-\bar{\gamma}^{e})(x^{e}-\bar{x}^{e})^{\mathrm{T}}\}=0,\ P_{13}^{e} \triangleq E\{(\gamma^{e}-\bar{\gamma}^{e})(X-\bar{X})^{\mathrm{T}}\}=0,\ P_{23}^{e} \triangleq E\{(x^{e}-\bar{x}^{e})(X-\bar{X})^{\mathrm{T}}\}=0 \quad \text{(B5c)}$$

$$P_{22}^{e} \triangleq P^{x}=E\{(x^{e}-\bar{x}^{e})(x^{e}-\bar{x}^{e})^{\mathrm{T}}\}=(v+b^{MSE})^{-1}(P\otimes V) \quad \text{(B5d)}$$

$$P_{33}^{e} \triangleq P^{X}=E\{(X-\bar{X})(X-\bar{X})^{\mathrm{T}}\}=\frac{(v-2d-2)tr(V)V+(v-2d)V^{2}}{(v-2d-1)(v-2d-2)^{2}(v-2d-4)} \quad \text{(B5e)}$$

where $b^{MSE}=s-d-sd-3$, and (B5c) can be easily calculated as (B5b). The results of (B5d) and (B5e) have been proven in Equation (65) and (66) of [6].

2) Moments of state variables with mixture distribution: the first two moments of X^{e} defined by (B1a) are

$$\bar{X}^{e,m} \triangleq E\{X^{e}\}=\int X^{e}\sum_{i=1}^{N}w_{i}\mathcal{GGIW}(\xi;\zeta_{i})\,\mathrm{d}X^{e}=\sum_{i=1}^{N}w_{i}\bar{X}_{i}^{e}=\sum_{i=1}^{N}w_{i}\begin{bmatrix}\left[\bar{\gamma}_{i},0_{1\times(d-1)}\right]\\ \left[\bar{x}_{i},0_{(sd)\times(d-1)}\right]\\ \bar{X}_{i}\end{bmatrix} \quad \text{(B6a)}$$

$$=\begin{bmatrix}\bar{\gamma}^{e,m}\\ \bar{x}^{e,m}\\ \bar{X}^{m}\end{bmatrix}=\sum_{i=1}^{N}w_{i}\begin{bmatrix}\left[\alpha_{i}/\beta_{i},0_{1\times(d-1)}\right]\\ \left[m_{i},0_{(sd)\times(d-1)}\right]\\ V_{i}/(v_{i}-2d-2)\end{bmatrix}=\begin{bmatrix}\left[\bar{\gamma}^{m}=\sum_{i=1}^{N}w_{i}\alpha_{i}/\beta_{i},0_{1\times(d-1)}\right]\\ \left[\bar{x}^{m}=\sum_{i=1}^{N}w_{i}m_{i},0_{(sd)\times(d-1)}\right]\\ \sum_{i=1}^{N}w_{i}V_{i}/(v_{i}-2d-2)\end{bmatrix} \quad \text{(B6b)}$$

$$P^{e,m} \triangleq E\{(X^{e}-\bar{X}^{e,m})(\cdot)^{\mathrm{T}}\}=\int(X^{e}-\bar{X}^{e,m})(\cdot)^{\mathrm{T}}\sum_{i=1}^{N}w_{i}\mathcal{GGIW}(\xi;\zeta_{i})\,\mathrm{d}X^{e} \quad \text{(B7a)}$$

$$=\sum_{i=1}^{N}w_{i}\int(X^{e}-\bar{X}_{i}^{e}+\bar{X}_{i}^{e}-\bar{X}^{e,m})(\cdot)^{\mathrm{T}}\mathcal{GGIW}(\xi;\zeta_{i})\,\mathrm{d}X^{e} \quad \text{(B7b)}$$

$$=\sum_{i=1}^{N}w_{i}\left[\int(X^{e}-\bar{X}_{i}^{e})(\cdot)^{\mathrm{T}}\mathcal{GGIW}(\xi;\zeta_{i})\,\mathrm{d}X^{e}+(\bar{X}_{i}^{e}-\bar{X}^{e,m})(\cdot)^{\mathrm{T}}\right] \quad \text{(B7c)}$$

$$=\sum_{i=1}^{N}w_{i}\left[P_{i}^{e}+(\bar{X}_{i}^{e}-\bar{X}^{e,m})(\cdot)^{\mathrm{T}}\right]\triangleq\begin{bmatrix}P^{\gamma,m} & P^{\gamma,x,m} & P^{\gamma,X,m}\\ (P^{\gamma,x,m})^{\mathrm{T}} & P^{x,m} & P^{x,X,m}\\ (P^{\gamma,X,m})^{\mathrm{T}} & (P^{x,X,m})^{\mathrm{T}} & P^{X,m}\end{bmatrix} \quad \text{(B7d)}$$

where

$$P^{\gamma,m} \triangleq \sum_{i=1}^{N} w_i \left[P_i^{\gamma} + (\bar{\gamma}_i - \bar{\gamma}^m)(\cdot)^{\mathrm{T}} \right]$$
$$P^{x,m} \triangleq \sum_{i=1}^{N} w_i \left[P_i^{x} + (\bar{x}_i - \bar{x}^m)(\cdot)^{\mathrm{T}} \right]$$
$$P^{X,m} \triangleq \sum_{i=1}^{N} w_i \left[P_i^{X} + (\bar{X}_i - \bar{X}^m)(\cdot)^{\mathrm{T}} \right]$$
$$P^{\gamma,x,m} \triangleq \sum_{i=1}^{N} w_i (\bar{\gamma}_i - \bar{\gamma}^m)(\bar{x}_i - \bar{x}^m)^{\mathrm{T}}$$
$$P^{\gamma,X,m} \triangleq \sum_{i=1}^{N} w_i \left[\bar{\gamma}_i - \bar{\gamma}^m, 0_{1\times(d-1)} \right] (\bar{X}_i - \bar{X}^m)^{\mathrm{T}}$$
$$P^{x,X,m} \triangleq \sum_{i=1}^{N} w_i \left[\bar{x}_i - \bar{x}^m, 0_{(sd)\times(d-1)} \right] (\bar{X}_i - \bar{X}^m)^{\mathrm{T}}$$

3) Moments of state variables with joint distribution: base on the above derivation, the first two moments of X^e defined by (B1b) are given by

$$\bar{X}^{e,1} = \begin{bmatrix} \bar{\gamma}^{e,1} \\ \bar{x}^{e,1} \\ \bar{X}^1 \end{bmatrix} = \begin{bmatrix} \left[\bar{\gamma}^1 = \bar{w}\alpha / \beta, 0_{1\times(d-1)} \right] \\ \left[\bar{x}^1 = \bar{w}m, 0_{(sd)\times(d-1)} \right] \\ \bar{w}V / (v - 2d - 2) \end{bmatrix} \tag{B8}$$

$$P^{e,1} = \bar{w}\left[P^e + (\bar{X}^e - \bar{X}^{e,1})(\cdot)^{\mathrm{T}} \right] \triangleq \begin{bmatrix} P^{\gamma,1} & P^{\gamma,x,1} & P^{\gamma,X,1} \\ (P^{\gamma,x,1})^{\mathrm{T}} & P^{x,1} & P^{x,X,1} \\ (P^{\gamma,X,1})^{\mathrm{T}} & (P^{x,X,1})^{\mathrm{T}} & P^{X,1} \end{bmatrix} \tag{B9}$$

Where

$$P^{\gamma,1} \triangleq \bar{w}\left[P^{\gamma} + (\bar{\gamma} - \bar{\gamma}^1)(\cdot)^{\mathrm{T}} \right]$$
$$P^{x,1} \triangleq \bar{w}\left[P^{x} + (\bar{x} - \bar{x}^1)(\cdot)^{\mathrm{T}} \right]$$
$$P^{X,1} \triangleq \bar{w}\left[P^{X} + (\bar{X} - \bar{X}^1)(\cdot)^{\mathrm{T}} \right]$$
$$P^{\gamma,x,1} \triangleq \bar{w}(\bar{\gamma} - \bar{\gamma}^1)(\bar{x} - \bar{x}^1)^{\mathrm{T}}$$
$$P^{\gamma,X,1} \triangleq \bar{w}\left[\bar{\gamma} - \bar{\gamma}^1, 0_{1\times(d-1)} \right] (\bar{X} - \bar{X}^1)^{\mathrm{T}}$$
$$P^{x,X,1} \triangleq \bar{w}\left[\bar{x} - \bar{x}^1, 0_{(sd)\times(d-1)} \right] (\bar{X} - \bar{X}^1)^{\mathrm{T}}$$

4) Moment matching: we match the first two moments calculated by (B1a) and (B1b) respectively, i.e., match (B6) with (B8) and (B7) with (B9). This yields

$$\bar{\gamma}^1 = \bar{\gamma}^m, \bar{x}^1 = \bar{x}^m, \bar{X}^1 = \bar{X}^m \tag{B10}$$

$$P^{\gamma,1} = P^{\gamma,m}, P^{x,1} = P^{x,m}, P^{X,1} = P^{X,m} \quad \text{(B11)}$$

$$P^{\gamma,x,1} = P^{\gamma,x,m}, P^{\gamma,X,1} = P^{\gamma,X,m}, P^{x,X,1} = P^{x,X,m} \quad \text{(B12)}$$

To match the moments, (B10), (B11), and (B12) must hold simultaneously. However, this cannot be achieved usually because of the inconsistency of these equations. So, careful approximations are needed. Here, we assume that the equations in (B12) are approximately true.

Solving the first equation in (B10) and the first equation in (B11) jointly, α and β can be calculated as

$$\alpha = \bar{\gamma}^m \beta / \bar{w}, \quad \beta = \frac{\bar{w}\bar{\gamma}^m}{\bar{w}P^{\gamma,m} - (1-\bar{w})^2 (\bar{\gamma}^m)^2} \quad \text{(B13)}$$

With the second equation in (B10), we can obtain

$$m = \bar{x}^m / \bar{w} \quad \text{(B14)}$$

Substituting the last equation in (B10) into the last equation in (B11) yields

$$\begin{aligned} v &= (1/2)\left[\hat{a} + (\hat{a}^2 - 8(\hat{b}+2))^{1/2}\right] + 2d \\ V &= (v - 2d - 2)\bar{X}^m / \bar{w} \end{aligned} \quad \text{(B15a)}$$

where $\hat{a} = \hat{b} + \hat{c} + 5$ with

$$\begin{aligned} \hat{b} &= \frac{[tr(\bar{X}^m)]^2}{\bar{w}tr(P^{X,m}) - (1-\bar{w})^2 tr[(\bar{X}^m)^2]} \\ \hat{c} &= \frac{tr[(\bar{X}^m)^2]}{\bar{w}tr(P^{X,m}) - (1-\bar{w})^2 tr[(\bar{X}^m)^2]} \end{aligned} \quad \text{(B15b)}$$

With the second equation in (B11), we have

$$\begin{aligned} P &= [p_{i,j}]^{s\times s}([p_{i,j}]^{s\times s})^{\mathrm{T}} \\ p_{i,j} &= (1/d)\sum_{l=1}^{d} q_{(i-1)d+l,(j-1)d+l} \end{aligned} \quad \text{(B16a)}$$

where

$$[q_{l,h}]^{sd\times sd}=[(v+b^{MSE})(I_s\otimes V^{-1/2})\hat{P}(I_s\otimes V^{-1/2})]^{1/2} \tag{B16b}$$

$$\hat{P}=P^{x,m}/\bar{w}-(1-\bar{w})^2 mm^{\mathrm{T}} \tag{B16c}$$

For the detailed derivation of (B16), see Equation (76)-(80) of [6]. All GGIW parameters are determined by (B13)-(B16). The full moment matching process is summarized in Table B1.

Table B1 Moment matching for component-merging

$$\sum_{i=1}^{N} w_i \mathcal{G}(\gamma;\alpha_i,\beta_i)\mathcal{N}(x;m_i,P_i\otimes X)\mathcal{IW}(X;v_i,V_i)\Rightarrow \bar{w}\mathcal{G}(\gamma;\alpha,\beta)\mathcal{N}(x;m,P\otimes X)\mathcal{IW}(X;v,V)$$

Input	$\bar{w},\{w_i,\alpha_i,\beta_i,m_i,P_i,v_i,V_i\}_{i=1}^{N}$
Measurement Rate	$\alpha=\bar{\gamma}^m\beta/\bar{w}$, $\beta=\bar{w}\bar{\gamma}^m/[\bar{w}P^{\gamma,m}-(1-\bar{w})^2(\bar{\gamma}^m)^2]$
	$\bar{\gamma}^m=\sum_{i=1}^{N} w_i\alpha_i/\beta_i$, $P^{\gamma,m}=\sum_{i=1}^{N} w_i[P_i^{\gamma}+(\bar{\gamma}_i-\bar{\gamma}^m)(\cdot)^{\mathrm{T}}]$, $\bar{\gamma}_i=\alpha_i/\beta_i, P_i^{\gamma}=\alpha_i/\beta_i^2$
Kinematic State	$m=\bar{x}^m/\bar{w}$, $P=[p_{i,j}]^{s\times s}([p_{i,j}]^{s\times s})^{\mathrm{T}}$
	$\bar{x}^m=\sum_{i=1}^{N} w_i m_i$, $p_{i,j}=(1/d)\sum_{l=1}^{d} q_{(i-1)d+l,(j-1)d+l}$ $[q_{l,h}]^{sd\times sd}=[\hat{v}(I_s\otimes V^{-1/2})\hat{P}(I_s\otimes V^{-1/2})]^{1/2}$ $\hat{v}=v+b^{MSE}$, $b^{MSE}=s-d-sd-3$, $\hat{P}=P^{x,m}/\bar{w}-(1-\bar{w})^2 mm^{\mathrm{T}}$ $P^{x,m}=\sum_{i=1}^{N} w_i[P_i^{x}+(\bar{x}_i-\bar{x}^m)(\cdot)^{\mathrm{T}}]$, $\bar{x}_i=m_i$, $P_i^{x}=(v_i+b^{MSE})^{-1}(P_i\otimes V_i)$
Extension State	$v=(1/2)[\hat{a}+(\hat{a}^2-8(\hat{b}+2))^{1/2}]+2d$, $V=(v-2d-2)\bar{X}^m/\bar{w}$
	$\hat{a}=\hat{b}+\hat{c}+5$, $\hat{b}=[tr(\bar{X}^m)]^2/\bar{P}^m$, $\hat{c}=tr[(\bar{X}^m)^2]/\bar{P}^m$ $\bar{P}^m=\bar{w}tr(P^{X,m})-(1-\bar{w})^2 tr[(\bar{X}^m)^2]$, $\bar{X}^m=\sum_{i=1}^{N} w_i\bar{X}_i$, $\bar{X}_i=V_i/(v_i-2d-2)$ $P^{X,m}=\sum_{i=1}^{N} w_i[P_i^{X}+(\bar{X}_i-\bar{X}^m)(\cdot)^{\mathrm{T}}]$, $P_i^{X}=\dfrac{(v_i-2d-2)tr(V_i)V_i+(v-2d)V_i^2}{(v_i-2d-1)(v_i-2d-2)^2(v_i-2d-4)}$

Output	$\{\alpha, \beta, m, P, v, V\}$
